# The “Loss” of Perineuronal Nets in Alzheimer's Disease: Missing or Hiding in Plain Sight?

**DOI:** 10.3389/fnint.2022.896400

**Published:** 2022-05-25

**Authors:** Jarrad M. Scarlett, Shannon J. Hu, Kimberly M. Alonge

**Affiliations:** ^1^Department of Medicine, University of Washington Medicine Diabetes Institute, Seattle, WA, United States; ^2^Department of Pediatric Gastroenterology and Hepatology, Seattle Children's Hospital, Seattle, WA, United States

**Keywords:** perineuronal nets, chondroitin sulfates, glycosaminoglycans, proteoglycans, Alzheimer's disease, extracellular matrix, mass spectrometry, immunohistochemistry

## Abstract

Perineuronal nets (PNNs) are chondroitin-sulfate glycosaminoglycan (CS-GAG) containing extracellular matrix structures that assemble around neurons involved in learning, memory, and cognition. Owing to the unique patterning of negative charges stemming from sulfate modifications to the attached CS-GAGs, these matrices play key roles in mediating glycan-protein binding, signaling interactions, and charged ion buffering of the underlying circuitry. Histochemical loss of PNN matrices has been reported for a range of neurocognitive and neurodegenerative diseases, implying that PNNs might be a key player in the pathogenesis of neurological disorders. In this hypothesis and theory article, we begin by highlighting PNN changes observed in human postmortem brain tissue associated with Alzheimer's disease (AD) and corresponding changes reported in rodent models of AD neuropathology. We then discuss the technical limitations surrounding traditional methods for PNN analyses and propose alternative explanations to these historical findings. Lastly, we embark on a global re-evaluation of the interpretations for PNN changes across brain regions, across species, and in relation to other neurocognitive disorders.

## Introduction

**Figure 1:**
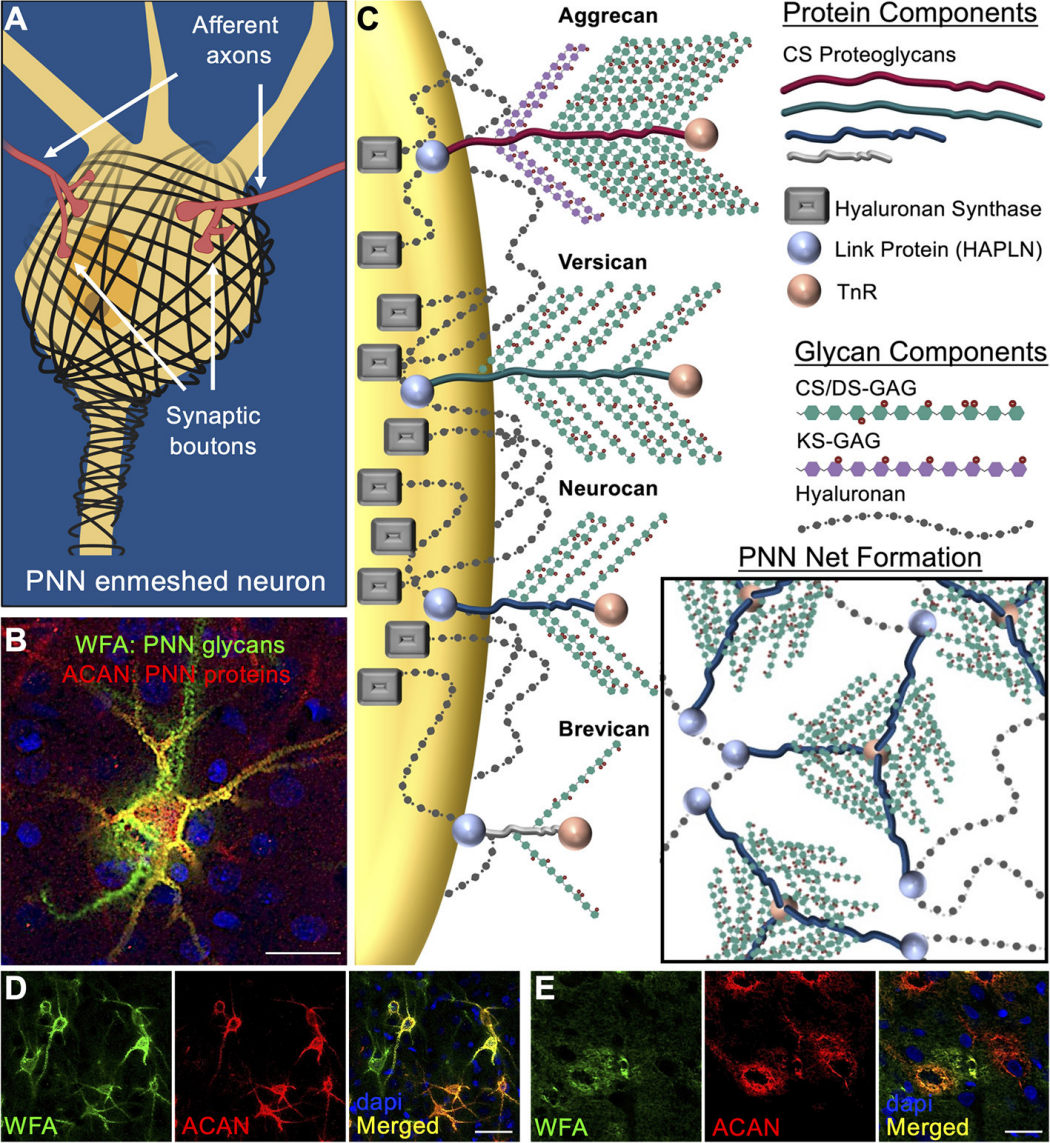
Perineuronal net composition and structure. **(A)** PNNs enmesh the soma and proximal axons and dendrites of target neurons (see Logsdon et al., 2021). Synaptic contacts between pre- and post- synaptic neurons are dynamically regulated by gaps formed in the PNN lattice. **(B)** Visualization of brain PNNs is possible by antibody labeling of the underlying CSPG core protein (aggrecan, *red*) and lectin labeling of the attached CS-GAGs (WFA, *green*) (rat motor cortex show) (see Alonge et al., 2020). **(C)** PNN matrices are primarily comprised of the lectin family of CSPG core proteins (e.g., aggrecan, versican, neurocan, and brevican). Link proteins attach the CSPGs to the underlying hyaluronan (HA) glycans, which are then anchored to the neuronal cell surface by hyaluronan synthase (HAS) proteins. PNN net formation occurs through tri-linkages between CSPGs and Tenascin R (TnR) glycoproteins in the extracellular space. PNNs in rat **(D)** visual cortex exhibits intricate, lattice-like formations surrounding the neuronal soma and extending processes of the enmeshed neuron, while rat **(E)** hypothalamus shows diffuse, unstructured matrix formation (see Alonge et al., 2020). PNN populations in both the cortex and hypothalamus exhibit heterogenous composition that includes WFA^+^/aggrecan^+^, WFA^+^/aggrecan^−^, and WFA^−^/aggrecan^+^ PNN substructures. Scale bar: **(B,E)** 25 μm, **(D)** 50 μm. WFA, *Wisteria floribunda* agglutinin; ACAN, aggrecan; PNN, perineuronal net; CS/DS-GAGs, chondroitin/dermatan sulfate-glycosaminoglycans; TnR, tenascin-R.

An emerging concept in the field of neurological disorders is that neurocircuit function is not dependent solely on the integrity of neurons, glia, and other brain cell types, but also heavily influenced by the surrounding extracellular matrix (ECM). Components of the ECM constitute ~20% of the brain's total volume (Nicholson and Syková, 1998) and growing evidence support a role of the brain's ECM as being one of the largest direct influencers of brain function. Perineuronal nets (PNNs) were first described by Camillo Golgi over a century ago as “delicate [pericellular] coverings mainly reticular in structure… of a continuous envelope that enwraps the body of all the nerve cells…” (Golgi, 1898; Vitellaro-Zuccarello et al., 1998). Further investigation showed that these PNN matrices exhibit a honeycomb structured ECM morphology that form condensed pericellular coats enmeshing the soma and proximal dendrites of key neurons in the brain (Figures 1A,B; see Alonge et al., 2020; Logsdon et al., 2021). The lattice-like formation characteristic of PNNs are produced by a combination of link protein (HAPLN) anchorage of PNN-associated chondroitin sulfate proteoglycans (CSPGs) onto the underlying neuron-attached hyaluronan (HA) and hyaluronan synthase (HAS) backbones, which are then molded into a net formation by Tenascin-R (TnR) cross-linkage of the CSPGs (Figure 1C). PNN variability is highly influenced by the incorporation of specific CSPGs into the matrix assemblies including traditional lectin CSPG family members aggrecan, versican, neurocan, and brevican (Figure 1C). Attached to the CSPG core proteins are variable quantities of chondroitin sulfate (CS) and dermatan sulfate (DS) glycosaminoglycan (GAG) chains, which consist of repeating disaccharide units of sulfated glucuronic acid (GlcA) (for CS) or iduronic acid (IdoA) (for DS) and *N*-acetylgalactosamine (GalNAc). Of noteworthy importance, aggrecan exhibits the greatest abundance of both CS/DS-GAG and keratan sulfate (KS)-GAG attached chains, while the other CSPG variants exhibit far fewer attachment sites. It is the abundance and composition of these glycan attachments that are predicted to underly PNN function by regulating extracellular protein-glycan interactions and cation buffering (Härtig et al., 1999; Smith et al., 2015). Although DS isomers are incorporated into the PNN CS/DS-GAG chains, genetic knockout of dermatan sulfate epimerase 2 (DS-epi2) in mice produces no histological PNN abnormalities or behavioral defects (Bartolini et al., 2012). Therefore, the remainder of this review will focus exclusively on the role of CS-GAGs in PNNs.

Histochemical detection of brain PNN structures has traditionally been accomplished by antibody labeling of the underlying CSPG core proteins and lectin labeling of the attached CS-GAGs, which have shown that PNNs exhibit both structured (Figure 1D; see Alonge et al., 2020) and diffuse (Figure 1E; see Alonge et al., 2020) matrix morphologies. Unlike other lectin CSPGs, aggrecan is found almost exclusively in PNNs and is therefore considered a key target for understanding the formation and function of these unique extracellular matrix structures (Yamada and Jinno, 2017). Histochemical labeling for both aggrecan and CS-GAGs (using *Wisteria floribunda* agglutinin (WFA) lectin) shows co-localization of these PNN components into WFA^+^/aggrecan^+^, WFA^−^/aggrecan^+^ and WFA^+^/aggrecan^−^ PNN structures (Figures 1D,E; see Alonge et al., 2020), which has been used to describe the region-specific heterogeneity of PNNs throughout the brain (Matthews et al., 2002; Yamada and Jinno, 2017; Miyata et al., 2018). However, additional work also suggests that PNN heterogeneity is driven by the underlying neuronal circuitry (Yamada and Jinno, 2017), as neurons themselves may directly regulate PNN composition by mediating both CSPG protein expression and the degree and composition of their CS-GAG attachments (Matthews et al., 2002).

As PNNs play key roles in forming and maintaining memory (Sorg et al., 2016; Testa et al., 2019; Carulli and Verhaagen, 2021), it is reasonable to ask whether patients diagnosed with neurocognitive and neurodegenerative disorders also develop pathological changes in the structure and/or composition of their brain PNN matrices. Alzheimer's disease (AD), which is the leading cause of progressive dementia worldwide, is a neurodegenerative disorder associated with the gradual decline in learning, memory, and cognition. The primary neuropathologic criteria for AD diagnosis is extracellular β-amyloid (Aβ) deposition and intracellular accumulation of hyperphosphorylated tau (pTau) (Braak and Braak, 1991). However, treatment outcomes for AD have not significantly improved for more than two decades despite the introduction of dozens of therapeutics targeting these neuropathologies (Mehta et al., 2017), suggesting the potential contribution of additional elements in the pathogenesis of AD. One increasingly attractive element are PNNs due to the growing evidence that changes in extracellular PNNs are tightly linked with AD neuropathology (Reichelt, 2020; Logsdon et al., 2021).

The objective of this hypothesis and theory article is to provide an in-depth analysis of established and emerging evidence linking brain PNN changes to AD. We will first highlight the known histological and compositional changes reported for PNNs identified in postmortem brain tissue from demented human patients and corresponding rodent models of AD neuropathology. We then review the accuracy of traditional histochemical methods for PNN analyses and highlight notable limitations in its replicability. Based on these findings, we then provide a novel theory describing PNN masking in the demented brain and propose new approaches to studying PNN changes in AD. Ongoing research that potentially links PNN changes to the AD pathogenesis may be key to furthering our understanding of this neuropathological disorder and guide the development of more effective therapeutic treatments.

## Evidence Linking Changes in Perineuronal Nets With Alzheimer's Disease

### PNN Histological Changes in Demented Postmortem Human Brain Tissue

**Figure 2:**
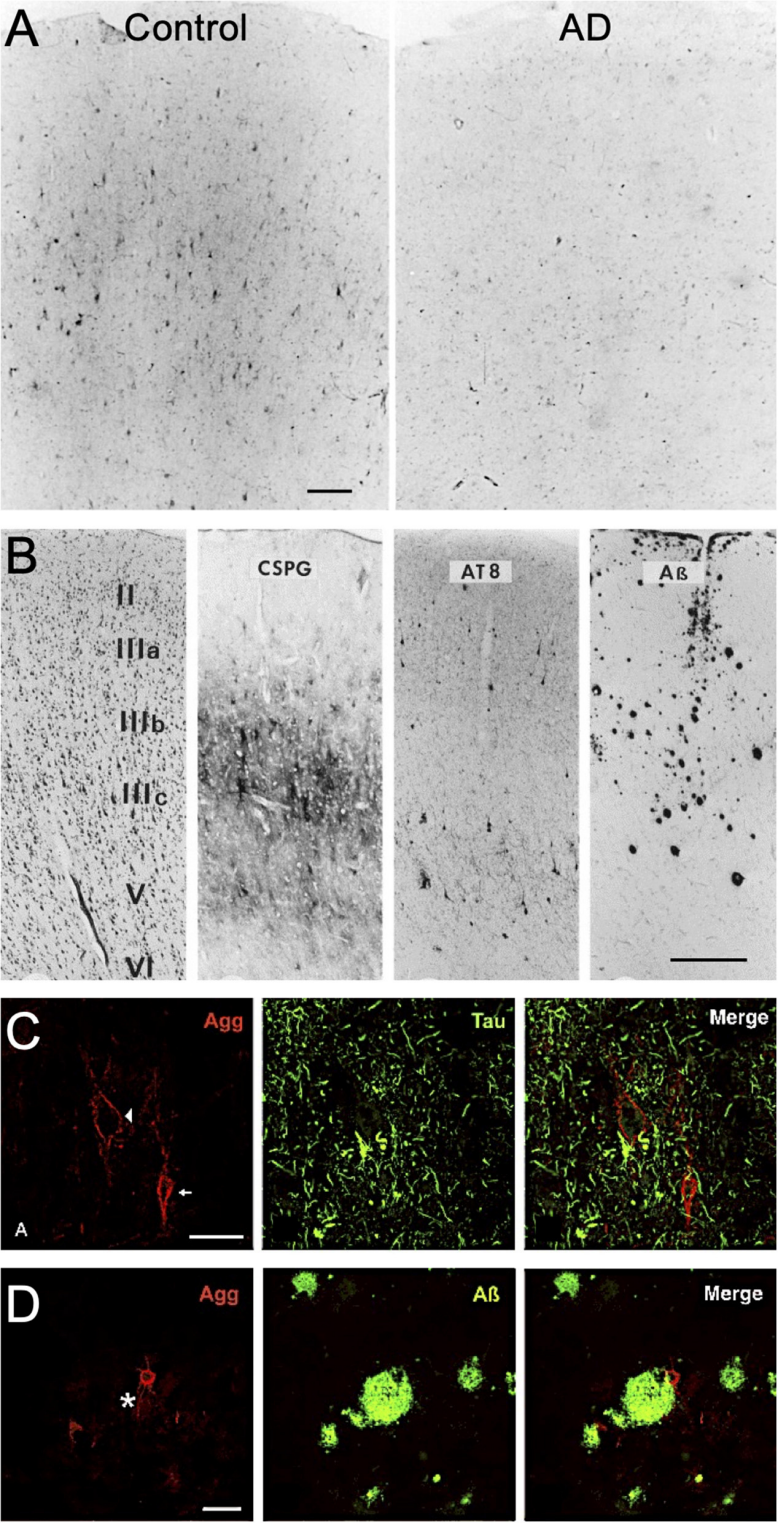
PNN changes in AD postmortem brain tissue. **(A)** *Vicia villosa* lectin labeling of PNN glycans in postmortem frontal cortex is decreased in patients with AD. Scale: 150 μm (see Kobayashi et al., 1989). **(B)** Laminar distribution of PNN CSPGs compared to AT8-labeling of pTau and ß-amyloid deposits in postmortem AD frontal cortex. Scale bar: 0.5 mm (see Brückner et al., 1999). Aggrecan^+^ PNNs are devoid of **(C)** pTau accumulation but show positive interactions with **(D)** ß-amyloid deposits (star indicates plaque location). Scale: 50 μm (see Morawski et al., 2010a). CSPG, chondroitin sulfate proteoglycan; Agg, aggrecan.

The historical use of lectins to identify PNN structures throughout the human brain have revealed consistent area-specific distribution patterns in the human cortex (Seeger et al., 1996; Hilbig et al., 2001). In contrast to normal brain tissue, studies investigating PNN changes in demented postmortem human brain tissue have shown disruptions in PNN integrity that associate with AD clinicopathology. Earlier studies using *Vicia villosa* (VVA) lectin staining of the PNN-attached CS-GAGs reported a significant reduction in the abundance of PNN glycan structures in the frontal cortex of postmortem AD brain (Figure 2A; see Kobayashi et al., 1989), which was subsequently recapitulated using *Wisteria floribunda* agglutinin (WFA) lectin staining (Table 1) (Baig et al., 2005). However, these results are in stark opposition to results generated from aggrecan or brevican labeling of the PNN CSPG core proteins in AD postmortem brain tissue, which showed either no change by immunohistochemistry (Brückner et al., 1999; Morawski et al., 2010a, 2012) or an increase in abundance by western analysis (Lendvai et al., 2013; Howell et al., 2015). Although these results collectively imply AD in humans is a glycan-specific disorder that is characterized by the selective loss of WFA^+^ CS-GAGs over aggrecan^+^ and brevican^+^ CSPGs, other studies have been unsuccessful in recapitulating these initial observations (Morawski et al., 2012; Crapser et al., 2020) (Table 1).

**Table 1:** PNN changes in postmortem Alzheimer's disease brain tissue.

**Brain region**	**PNN changes**	**AD Neuropathology**	**References**
Frontal and Temporal Cortices	↓ PNN glycans (*Vicia villosa* lectin)	Not investigated	Kobayashi et al., 1989
Motor, Auditory, Temporal, Prefrontal, Cingulate, and Entorhinal cortices	PNN core proteins unchanged (CSPG AB1019 antibody)	pTau^+^ neurons lack PNNs	Brückner et al., 1999
Frontal Cortex	↓ PNN glycans (*Wisteria floribunda* agglutinin lectin)	No correlation between number of PNNs and pTau, Aβ, or microglia	Baig et al., 2005
Insular Cortex, Claustrum, Amygdala, Striatum, Basal Forebrain, Thalamus, Tuberomammillary Nucleus, Mammillary Body, Substantia nigra, Oculomotor Complex, Rostral Interstitial Nucleus, Dorsal Raphe Nucleus, Lateral Parabrachial nucleus, Locus Coeruleus, Pontine Reticular formation	PNN core proteins unchanged (aggrecan 7D4 antibody)	• pTau^+^ neurons lack PNNs • PNN distribution independent to Aβ	Morawski et al., 2010a
Occipital and Temporal Cortices	• PNN glycans unchanged (*Wisteria floribunda* agglutinin lectin) • PNN core proteins unchanged (aggrecan 7D4 antibody and brevican B50 antibody)	• pTau^+^ neurons lack PNNs • PNN distribution independent to Aβ	Morawski et al., 2012
Hippocampus	↑ PNN core proteins (brevican B50 antibody)	pTau^+^ neurons lack PNNs	Lendvai et al., 2013
Superior Frontal Gyrus	↑ PNN core proteins (brevican sc-20555 antibody)	Not investigated	Howell et al., 2015
Middle Frontal Gyrus	↓ PNN core proteins (aggrecan 7D4 antibody)	Interaction between Aβ and PNNs	Crapser et al., 2020

*PNNs, perineuronal nets; CSPG, chondroitin sulfate proteoglycan*.

In contrast to the ongoing controversy regarding the nature of changes in WFA/CS-GAG^+^ and aggrecan/CSPG^+^ PNN subtypes in postmortem AD brain tissue, a more broadly accepted pattern describing the spatial distribution of AD neuropathology and PNN matrices has emerged. Whereas, there appears to be no significant overlap between extracellular Aβ deposits and PNN distribution in AD brain tissue, neurons ensheathed by either WFA^+^ or aggrecan^+^ PNNs remain devoid of pTau accumulation (Figure 2B; see Brückner et al., 1999). These findings have given rise to the hypothesis that neurons that are surrounded by PNNs are “protected” from neurofibrillary tangle formation (Brückner et al., 1999; Morawski et al., 2010a, 2012). Combined with histological findings that stable PNNs are largely absent surrounding pTau^+^ neurons (Figure 2C; see Morawski et al., 2010a), but exist in the presence of Aβ deposits (Figure 2D; see Morawski et al., 2010a), these observations provide a strong rationale to support additional studies that further explore mechanisms driving the complex relationship between PNNs and pTau accumulation in AD.

Although the formation of PNNs does not appear to be disrupted by Aβ deposits (Figure 2D; see Morawski et al., 2010a), we acknowledge the potential for PNN interactions with the adjacent plaques. Histological staining for different CS isomers in human AD brain tissue, including both non-sulfated and sulfated variants, showed positive staining surrounding the periphery of senile plaque deposits but were not detected within the plaque cores themselves (Dewitt et al., 1993). Since sulfated GAGs have been shown to exhibit high binding affinity to Aβ (Ariga et al., 2010), the close interactions observed between CS-GAGs and Aβ deposits may result in enhance Aβ fibril formation within these regions. Additional studies are needed to determine if the source of these CS-Aβ interactions stem from changes in traditional PNNs or from additional CS-GAG-rich matrices residing in the brain including glial scars (Mckeon et al., 1999; Gilbert et al., 2005; Alonge et al., 2021).

### PNN CS-GAG Compositional Changes in Demented Postmortem Human Brain Tissue

**Figure 3:**
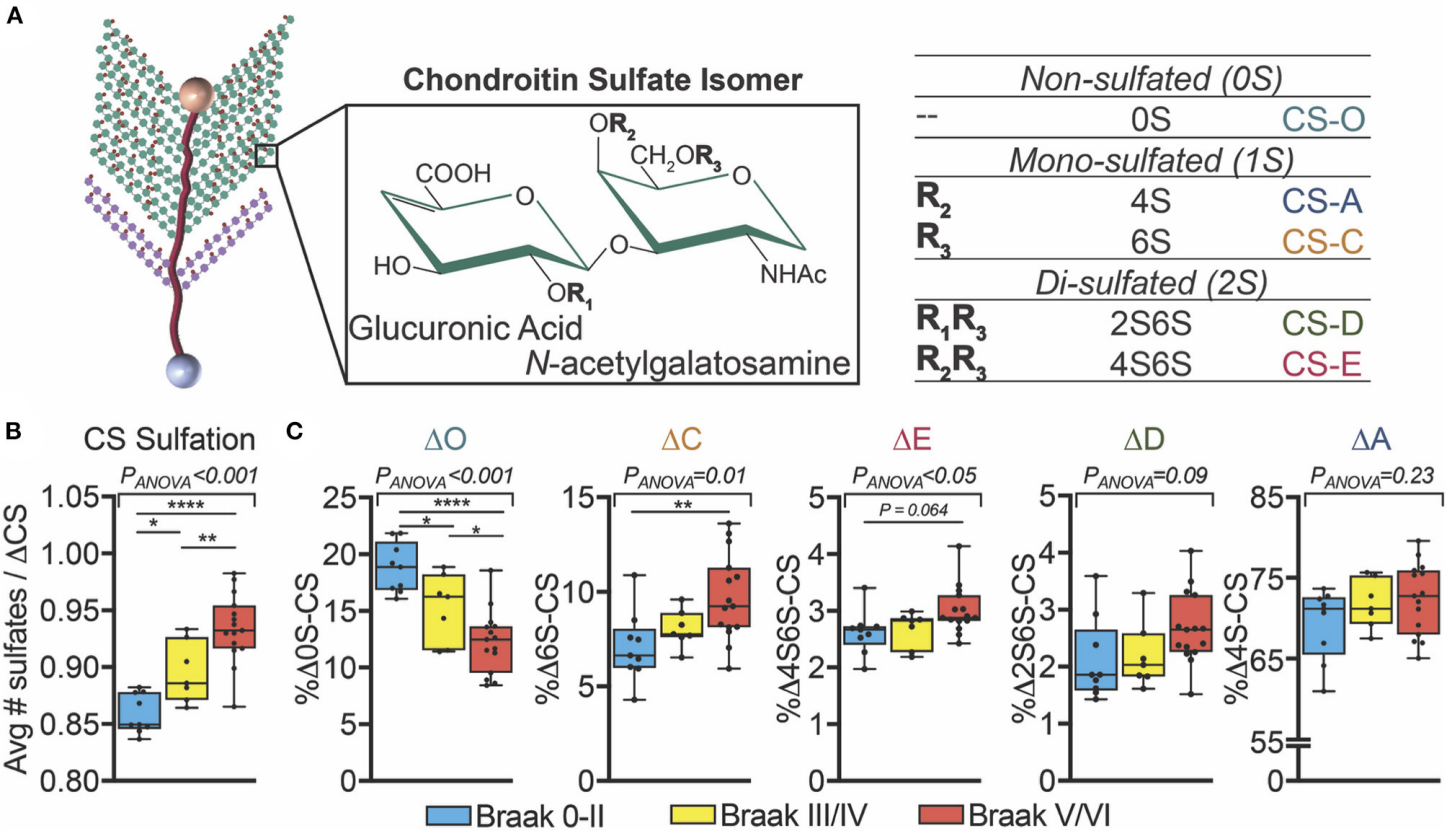
Changes in human AD brain CS-GAG sulfation patterns correlate to Braak stage. **(A)** PNN attached CS-GAGs are comprised of five key CS isomers including CS-O (0S), CS-A (4S), CS-C (6S), CS-D (2S6S), and CS-E (4S6S), which differ by the number of sulfation attachments (non- (0S), mono- (1S), and di- (2S) sulfated variants). The relative abundance of each isomer incorporated into the PNN matrix comprises a “CS-GAG sulfation code” that can influence both PNN stability and function. **(B,C)** Demented (AD) and non-demented (controls) were separate by Braak pathology including Braak 0-II (*blue*, controls), Braak III/IV (*yellow*, controls), and Braak V/VI (*red*, AD) (see Logsdon et al., 2021). **(B)** Hypersulfation of the CS-GAGs and changes in **(C)** the relative abundance of each CS isomer isolated from the middle frontal gyrus correlates to Braak stage (see Logsdon et al., 2021). *p < 0.05, **p < 0.01, ****p < 0.0001. CS, chondroitin sulfate.

Inconsistent with the historical evidence of reduced PNN labeling of CS-GAGs in postmortem AD brain tissue (Kobayashi et al., 1989; Baig et al., 2005), multiple reports have shown that the total abundance of brain CS-GAGs remains unchanged (Shimizu et al., 2009; Huynh et al., 2019; Logsdon et al., 2021). Instead of total abundance, more recent data demonstrate that changes in sulfated GAGs in AD brain tissue are associated with altered glycan-protein binding with extracellular factors, including decreased interactions with FGF2 and VEGF and increased binding to Tau (Huynh et al., 2019). These shifts in glycan-protein interactions are predicted to be driven primarily by changes in the PNN CS-GAG isomer composition, which consists of five differentially sulfated CS isomers in the human brain including non-sulfated [CS-O (0S)], mono-sulfated [CS-A (4S), CS-C (6S)] and di-sulfated [CS-D (2S6S), CS-E (4S6S)] variants (Figure 3A). The relative abundance of each CS isomer incorporated into the PNN matrix comprises a matrix “sulfation code” that links glycan-protein interactions with PNN biological functions including neuroplasticity and synaptic stability [CS-A (4S) and CS-C (6S) isomers (Miyata et al., 2012; Foscarin et al., 2017; Yang et al., 2021)], diffusion [CS-O (0S) (Syková and Nicholson, 2008)], neuroregeneration [CS-D (2S6S) (Shida et al., 2019)], and neuroinflammation [CS-E (4S6S) (Gilbert et al., 2005)]. We refer to recently published reviews that provide detailed and specific descriptions of glycan-protein binding interactions including that of CS-GAGs with extracellular growth factors, receptors, and guidance proteins (Djerbal et al., 2017; Vallet et al., 2021). Changes in the underlying PNN CS-GAG sulfation code (and thus altered glycan-protein interactions) has been implicated in promoting long-term neurocircuit dysfunction that is associated with AD.

In agreement with this glycan-centric perspective of AD, our lab recently reported striking differences in CS-GAG sulfation patterns in the middle frontal gyrus of human postmortem AD brain tissue compared to non-demented controls (Logsdon et al., 2021). Our analysis revealed that CS-GAGs isolated from the demented brain are hypersulfated (Figure 3B; see Logsdon et al., 2021), characterized by a significant decrease in the non-sulfated CS-O (0S) isomer incorporation and corresponding increases in the sulfated CS-C (6S), CS-D (2S6S), and CS-E (4S6S) isomers (Figure 3C; see Logsdon et al., 2021). Consistent with previously published reports describing PNN distribution surrounding AD neuropathology in the human brain (Figures 2C,D; see Morawski et al., 2010a), these CS-GAG sulfation pattern changes were not associated with the appearance of Aβ deposits (*unpublished observations*) but were positively correlated with the degree of pTau accumulation (Logsdon et al., 2021). Importantly, we observed that changes in brain CS-GAG sulfation patterns were present in non-demented postmortem brain tissue *before* the appearance of AD clinicopathology, and that these changes significantly correlated to cognitive function in these individuals (Logsdon et al., 2021). The progressive shift in CS-GAG sulfation patterns prior to AD clinicopathology appears to occur in parallel with a gradual increase in brevican CSPG core protein expression in mildly cognitive impaired (MCI) and AD brain tissue (Howell et al., 2015). As such, we interpreted these early changes in PNN glycan and protein remodeling events as novel neuropathological changes stationed upstream of AD diagnosis and appearance of toxic neuropathology that further supports the theory that AD is, at least in part, a glycan-driven neurological disorder.

The finding that CS-GAG sulfation patterns (and not abundance *per se*) is altered in postmortem AD brain tissue is in agreement with a recent report that identified changes in “chondroitin sulfate dermatan sulfate metabolism (reactome)” as a key gene set underlying the vulnerability of pTau accumulation and neurodegeneration in human AD brain tissue (Grothe et al., 2018). Positively enriched genes include those involved in the production of CSPG core proteins (e.g., neurocan, brevican) and CS-GAG biosynthesis and sulfate modifications (e.g., CHST7, CHSY3). Increased CHST7 gene expression, which drives expression of mono-sulfated CS-C (6S) isomer, agrees with our finding for the increased abundance of CS-C by mass spectrometry (Logsdon et al., 2021). Meanwhile, the shift in PNN-CSPG expression from aggrecan to neurocan and brevican implies that AD may also associate with alterations in the underlying core protein expression distinct from traditional PNN CSPG content. This is of great importance to consider when interpreting studies that report reduced PNN content in AD brain tissue through the loss of aggrecan core protein staining, which is discussed in greater detail later in this article.

In mice, the absence of WFA^+^ PNN structures in early neurodevelopment (Carulli et al., 2010; Lensjø et al., 2017) also coincide with the establishment of a CS-C (6S) isomer-rich “developmental CS-GAG sulfation code” (Miyata et al., 2012; Alonge et al., 2019), which is predicted to prevent premature PNN formation during this critical period (Miyata and Kitagawa, 2016). Corresponding to the increase in CS-C (6S) expression, the developing brain also exhibits strong expression of the neurodevelopmental CSPG, neurocan, over that of the mature CSPG variants (Carulli et al., 2010). The establishment of stable WFA^+^ PNNs is not apparent until “critical periods” of brain development are reached; here, developmental CS-GAG patterns switch from a CS-C (6S) isomer-rich patterning to a CS-A (4S) isomer-rich “adult CS-GAG sulfation code” in association with the decrease in developmental neurocan expression and appearance of mature CSPGs including aggrecan, versican, and brevican (Carulli et al., 2010). Moreover, recent evidence suggest that overabundance of the mature CS-A (4S) isomer in adulthood may play a key role in mediating age-related cognitive decline by preventing experience-driven circuit plasticity and limiting new memory formation (Foscarin et al., 2017). In contrast to normal age-related changes in PNNs and CS-GAG sulfation patterning, the human AD brain exhibits a paradoxical increase the developmental CS-C (6S) isomer (Logsdon et al., 2021) and neurocan core protein expression (Grothe et al., 2018). Together, these findings suggest that the demented human brain exhibits PNN and CS-GAG characteristics more commonly associated with neurodevelopment and future studies are thus warranted to understand this perplexing relationship.

### PNN Changes in Mouse Models of AD Neuropathology

Rodent models of AD neuropathology exhibit variable changes in brain PNN abundance and composition, many of which appear to be age- and strain-dependent. Due to the direct interactions between CS-GAGs and Aβ deposits (Figure 2D; see Morawski et al., 2010a), the most studied mouse models of PNN interactions with AD pathology are that of amyloidosis (Ariga et al., 2010). Although postmortem human AD brain tissue shows a significant decrease in the abundance of WFA^+^ PNN matrices, one of the earliest PNN characterization studies in the APP/PS1 mouse model of Aβ overexpression showed a significant *increase* in hippocampal WFA^+^ labeling of parvalbumin neurons as well as a corresponding *increase* in PNN protein abundance (i.e., neurocan, brevican, link protein, TnR) (Table 2) (Végh et al., 2014). In contrast to this finding, two additional mouse lines of Aβ overexpression, Tg2576/APPsw and APP^NL−F^ mice, showed no change in hippocampal PNN abundance or distribution compared to controls (Morawski et al., 2010b; Sos et al., 2020). Further, the abundance of amyloid plaque formation and degree of gliosis in Tg2576/APPsw mice occurred independently of the density and laminar distribution of the PNNs themselves (Morawski et al., 2010b). A more recent study using the same Tg2576/APPsw mouse line reported a slightly different outcome; whereas Morawski et al. reported no change in PNN distributions, Rey and colleagues reported a significant *reduction* in hippocampal PV^+^ and PV^+^/WFA^+^ neurons in this mouse line (Rey et al., 2022). This most recent report agrees with a similar study that also showed a significant reduction in PV^+^ and PV^+^/WFA^+^ neurons, in addition to decreased WFA mean fluorescence intensity, within select hippocampal subregions of Tg2576/APPsw mice as early as 3 months of age (Cattaud et al., 2018). Since Tg2576/APPsw mice fail to exhibit parenchymal Aβ deposit until 11–13 months of age (Hsiao et al., 1996), the loss of WFA^+^ PNNs reported in the latter two studies occurred months prior to, and independent of, the development of Aβ fibril formation in this mouse line.

**Table 2:** PNN Changes in mouse models of AD neuropathology.

**Mouse model (age tested)**	**AD neuropathology**	**Effects on PNNs**	**References**
APP/PS1 (1.5–12 months)	Model of amyloidosis	↑ WFA^+^ PNNs	Végh et al., 2014
Tg2576 (APPsw) ^a^ (14–18 months) ^b^ (3–15 months) ^c^ (15–16 months) ^d^ (9–10 months)	Model of amyloidosis	^a^ No change in WFA^+^ or aggrecan^+^ PNNs ^b^ ↓ PV^+^/WFA^+^ PNNs ^c^ ↓ CS-GAGs on brevican ^d^ ↓ PV^+^/WFA^+^ PNNs	^a^ Morawski et al., 2010b ^b^ Cattaud et al., 2018 ^c^ Ajmo et al., 2010 ^d^ Rey et al., 2022
APP^NL−F^ (21-month)	Model of amyloidosis	No change in WFA^+^ PNNs	Sos et al., 2020
5xFAD (4–18 months)	Model of amyloidosis	↓ WFA^+^ PNNs	Crapser et al., 2020
3xTg-AD (4–18 months)	Model of amyloidosis and tauopathy	↓ WFA^+^ PNNs	Javonillo et al., 2021
P301S (3 months)	Model of tauopathy	No change in WFA^+^ PNNs	Yang et al., 2015

a−d *Corresponds to listed references; WFA, Wisteria floribunda agglutinin; PNNs, perineuronal nets; PV, parvalbumin; CS-GAGs, chondroitin sulfate-glycosaminoglycans*.

The observation that WFA^+^ PNN CS-GAG labeling is reduced in Tg2576/APPsw mice conflicts with earlier reports describing no significant changes in the distribution of aggrecan^+^ PNN CSPG core protein. One potential explanation for this discrepancy may be provided by a fourth study in Tg2576/APPsw mice that showed a significant decrease in hippocampal CS-GAG attachments (but not core protein loss) of the PNN CSPG brevican (Ajmo et al., 2010). Taken together, the loss of CS-GAG attachments to CSPG core proteins and the reduction in WFA^+^ PNN labeling in the absence of total aggrecan or brevican core protein changes suggest that Tg2576/APPsw mice may undergo PNN remodeling, potentially involving CSPG deglycosylation and/or shifts in the expression of the underling CSPG protein expression, prior to the accumulation of Aβ.

Similar to Tg2576 mice, 4 month-old 5xFAD mice overexpressing Aβ exhibit a significant decrease in WFA^+^ PNNs in the cortex and subiculum (Crapser et al., 2020). Although the loss of WFA^+^ PNNs occurred in the presence of Aβ fibril formation in these mice, restoration of PNNs using the microglia inhibitor, PLX5622, was achieved even in the absence of altered Aβ plaque load, suggesting that the effect of PLX5622 to increase WFA^+^ PNNs in 5xFAD mice was due to direct inhibition of microgliosis and independent of Aβ formation *per se*. This finding agrees with recent results obtained in the 3xTg-AD mouse model of AD neuropathology that expresses both Aβ and neurofibrillary tau tangle formation. In this study, 18 month-old 3xTg-AD mice also displayed a significant reduction in WFA^+^ PNNs in the cortex and subiculum in association with increases in Aβ, pTau, and gliosis compared to normal controls (Javonillo et al., 2021). Importantly, restoration of WFA^+^ PNNs through PLX5622-driven inhibition of microgliosis also occurred independently to changes in Aβ plaque load (notably, pTau expression was not analyzed) (Crapser et al., 2020). Although it remains unclear whether microglia inhibition in 5xFAD and 3xTg-AD mice restores PNNs to that of wild-type control levels, the outcomes of these studies nevertheless complement recent discoveries that microglia play critical roles in daily PNN maintenance and turnover, since inhibition of microglia in normal mouse brain also increases WFA^+^ PNN abundance (Liu et al., 2021; Barahona et al., 2022).

While PNN enmeshed neurons appear to exhibit resilience to pTau accumulation (Figure 2C; see Morawski et al., 2010a), few studies have investigated the relationship between PNN changes in mouse models of tauopathy. One study using the Tg301S mouse model of pTau overexpression showed no difference in the number of perirhinal cortex WFA^+^ PNNs compared to controls (Yang et al., 2015). Interestingly, Chondrotinase ABC (ChABC) enzymatic digestion of PNN CS-GAGs resulted in memory restoration in Tg301S mice 7 days post treatment without influencing pTau accumulation, effects that were lost 25 days post treatment after CS-GAG abundance returned to normal. Collectively, these findings demonstrate that (1) memory restoration is possible even in the presence of pathological pTau, and (2) that ChABC digestion of PNN CS-GAGs positively influences memory recovery in mouse models of tauopathy. These results also imply that underlying mechanisms driving pTau-associated memory deficits may be mediated through direct changes in the PNN CS-GAG sulfation patterning and corresponding glycan-protein interactions with the nearby neuropathology.

## Selectivity of WFA Lectin Labeling of Brain Perineuronal Nets

The inconsistency between the histochemical loss of PNNs in postmortem human AD brain tissue and the absence of changes in total CS-GAG abundance now raises two new possibilities: (1) that changes in the species of the underlying CSPG core protein (and resulting number of CS-GAG attachment sites) underlies potential changes in PNN labeling in the brain, and/or that (2) that WFA lectin labeling of PNN CS-GAGs is significantly influenced by the CS-GAG sequence such that the loss of WFA^+^ PNN labeling in AD postmortem brain tissue is driven by changes in the underlying CS-GAG sulfation composition independent of changes in total PNN abundance *per se*.

### WFA and Cat-316 Selectively Bind to the Non-sulfated CS-O (0S) Isomer

**Figure 4:**
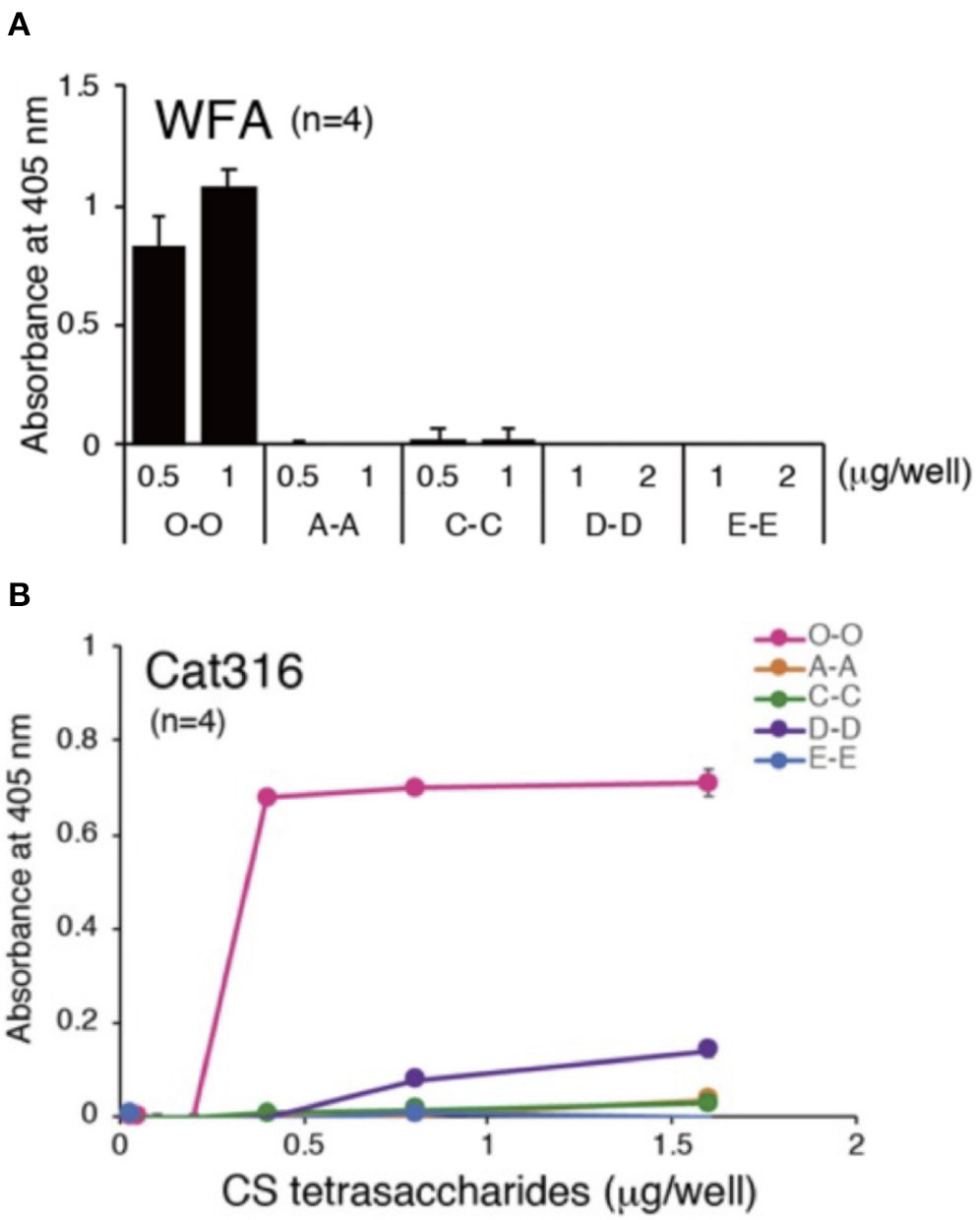
PNN CS-GAG recognizing lectins and antibodies bind to the non-sulfated CS-O (0S) isomer. Both **(A)** WFA and **(B)** Cat-316 PNN glycan-recognizing antibodies specifically interact with non-sulfated CS-O (0S) tetrasaccharides (see Nadanaka et al., 2020). Chemically synthesized biotinylated tetrasaccharides (O-O, A-A, C-C, D-D, and E-E) were immobilized onto streptavidin-coated plates and binding affinity of WFA and Cat-316 to the different CS tetrasaccharides were measured by ELISA. CS, chondroitin sulfate; WFA, *Wisteria floribunda* agglutinin.

In support of the latter theory, an elegant study published by Nadanaka et al. measured WFA lectin (labels CS-GAGs from all CSPGs) (Figure 4A; see Nadanaka et al., 2020) and Cat-316 antibody (labels CS-GAGs from aggrecan) (Figure 4B; see Nadanaka et al., 2020) interaction differences between all non-, mono-, and di-sulfated CS isomer tetrasaccharides. Results from this study clearly demonstrated that both WFA and Cat-316 preferentially recognize non-sulfated CS-O (0S) tetrasaccharide units relative to sulfated isomer variants. A notable limitation of these *in vitro* studies is the use of synthesized CS oligosaccharides, and we acknowledge that interaction between WFA and/or Cat-316 to longer, more complex endogenous CS-GAG chains may greatly influence binding properties of these labels. Nevertheless, the results from this study do provide strong evidence that WFA and Cat-316 should not be thought of as universal, non-specific histological labels for PNNs and aggrecan, respectively, but instead are highly influenced on the composition and abundance of CS-O (0S) units incorporated into the PNN associated CS-GAG chains. We now hypothesize that the selective binding of WFA to the non-sulfated CS-O (0S) unit is not only likely to influence the efficiency of WFA and Cat-316 to label PNNs in normal brain tissue but also highly susceptible to mislabeling of PNNs under diseased conditions associated with CS-GAG hypersulfation, as observed in postmortem AD brain tissue (Figure 3; see Logsdon et al., 2021). In summary, the finding that WFA and Cat-316 preferentially bind to the non-sulfated CS-O (0S) isomer now justifies a re-interpretation of lectin PNN labeling studies both in mouse and human tissues.

### WFA^+^ PNN Labeling Is Lost in Mice With Reduced CS-O (0S) Expression

**Figure 5:**
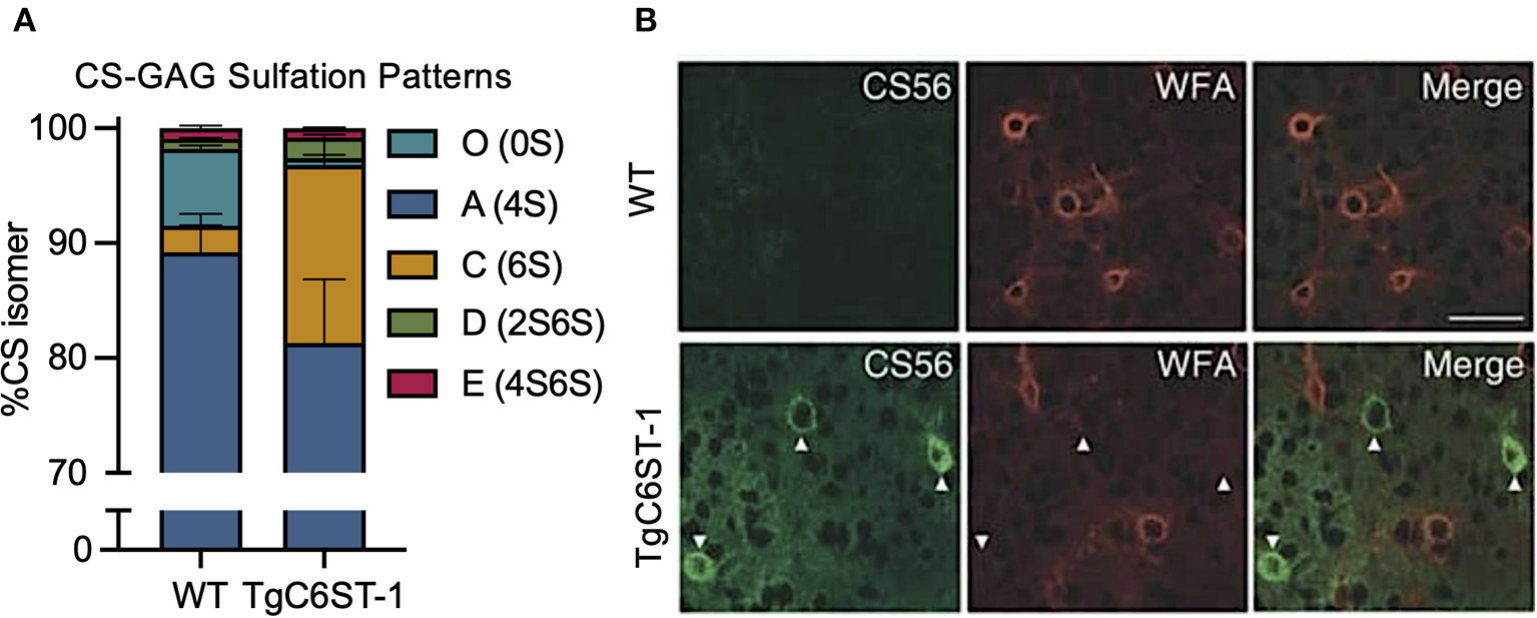
Overexpression of the CS-C (6S) isomer alters PNN CS-GAG sulfation patterns and histochemical detection. TgC6ST-1 mice overexpressing the CS-C (6S) isomer exhibit **(A)** increased mono-sulfated CS-C (6S) and decreased non-sulfated CS-O (0S) isomer expression (see Miyata et al., 2012). **(B)** Histochemical labeling of brain PNNs shows decreased WFA^+^ PNN structures in association with increased CS56^+^ PNN labeling in TgC6ST-1 mice compared to controls. Scale: 50 μm (see Miyata et al., 2012). CS-GAGs, chondroitin sulfate-glycosaminoglycans; WFA, *Wisteria floribunda* agglutinin.

One such study poised for re-interpretation is the reported loss of WFA^+^ PNNs in the C6ST-1 (CHST3) transgenic mouse model of CS-C (6S) overexpression (Miyata et al., 2012). Overproduction of the sulfated CS-C (6S) isomer in these transgenic mice resulted in a dramatic increase in the expression of the mono-sulfated CS-C (6S) isomer at the expense of greatly reducing the abundance of the non-sulfated CS-O (0S) isomer (Figure 5A; see Miyata et al., 2012). Corresponding to the reduction in CS-O (0S), these mice also exhibited a significant loss of WFA^+^ PNNs surrounding GABAergic neurons in the visual cortex compared to littermate controls (Figure 5B; see Miyata et al., 2012). However, the loss of WFA^+^ PNNs was supplemented by the appearance of CS56^+^ PNNs, which specifically labels CS-GAGs rich in CS-C (6S) (Figure 5B; see Miyata et al., 2012). Additional characterization of these TgC6ST-1 mice showed that the total abundance of the aggrecan core protein remained unchanged compared to controls (Miyata et al., 2012). Thus, the reduction of WFA^+^ PNNs observed in the TgC6ST-1 mice was not necessarily due to loss of PNNs *per se*, but may have represented the formation of new PNN subtypes containing altered CS sulfation patterning unrecognized by traditional WFA lectin labeling. Combined with the newly acquired knowledge that WFA definitively recognizes and binds to CS-O (0S) isomers (Figure 4; see Nadanaka et al., 2020), we now predict that the specific reduction of the CS-O (0S) isomer may have directly influenced the corresponding loss of WFA^+^ PNN labeling in TgC6ST-1 mice, with the overexpression of CS-C (6S) potentially secondary to this effect.

### Brain Regions Containing High CS-O (0S) Isomer Abundance Exhibit Increased WFA^+^ PNN Labeling

**Figure 6:**
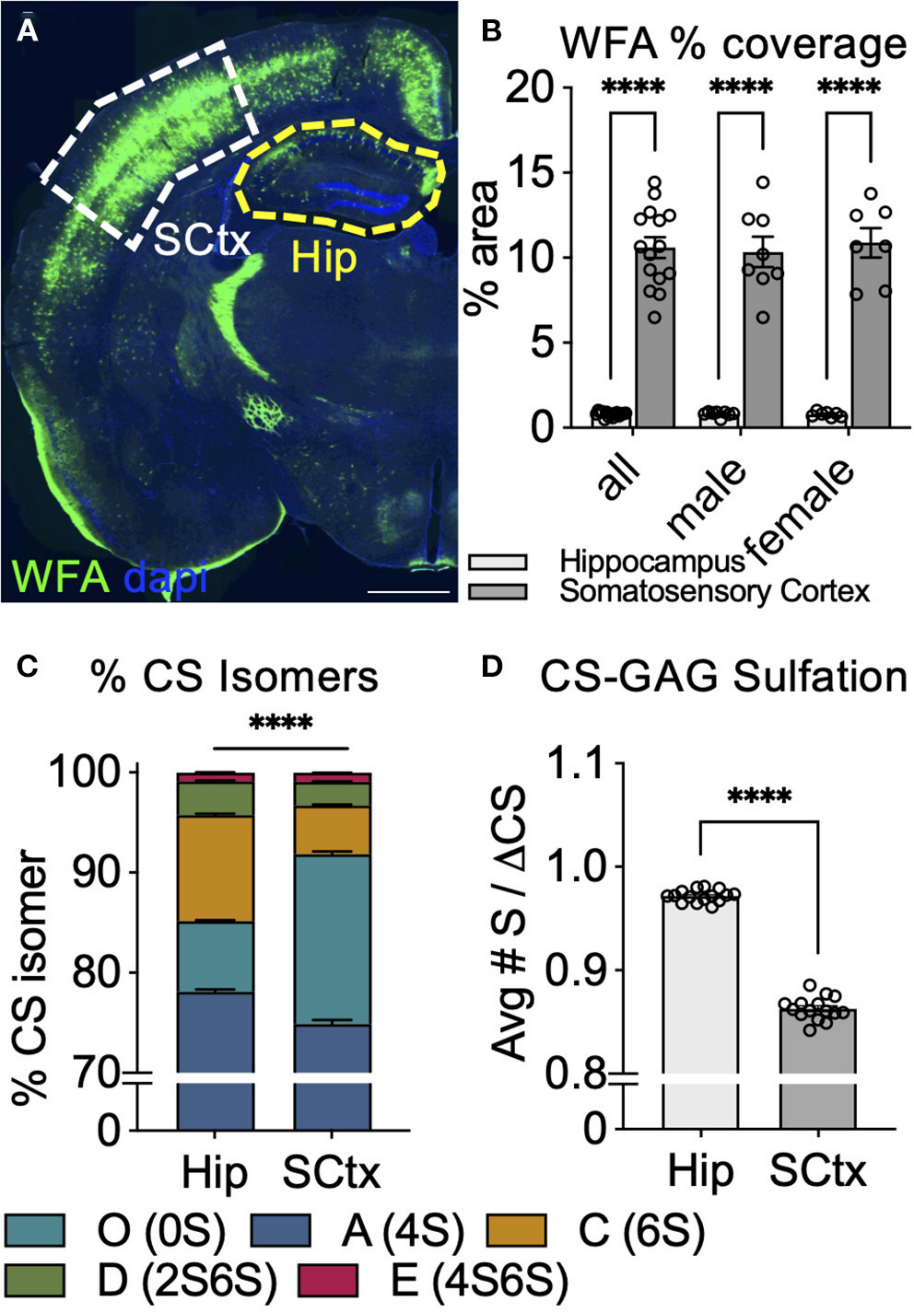
Abundance of WFA^+^ PNNs associates with changes in CS-GAG sulfation patterns across brain regions. **(A,B)** WFA labeling of PNNs in adult mouse brain shows increased WFA^+^ PNNs in the somatosensory cortex (SCtx, *white*) and decreased WFA^+^ PNNs in the adjacent hippocampus (Hip, *yellow*) (*n* = 8 male, *n* = 7 female mice) Scale: 1 mm. Interregional differences in WFA^+^ PNN-labeling significantly associate with **(C)** decreased abundance of the non-sulfated CS-O (0S) isomer and **(D)** hypersulfation of CS-GAGs. ****p < 0.0001. CS-GAG, chondroitin sulfate-glycosaminoglycan; WFA, *Wisteria floribunda* agglutinin; SCtx, somatosensory cortex; Hip, hippocampus.

If WFA labeling of PNNs is indeed directly dependent on the abundance of CS-O (0S) incorporated into the CS-GAG chains (Figures 4, 5), we would predict that variations in WFA labeling would be observed naturally across brain regions depending on local differences in CS-GAG sulfation patterns. As a first step to test this hypothesis, we stained for PNNs using WFA in adult male and female PFA-fixed mouse brain tissue (Figure 6A; Supplementary Methods). From this stain, we noted that the somatosensory cortex (SCtx) exhibited a 13.3-fold (*p* < 0.0001) increase in WFA % area coverage compared to the adjacent hippocampal (Hip) subregion (Figure 6B). To determine whether this effect may have been influenced by changes in WFA binding affinity to the local CS-GAG chains, we manually dissected both the SCtx and Hip from the same set of fixed tissues for CS isomer extraction and quantitative analysis using mass spectrometry as previously described (Supplementary Methods) (Alonge et al., 2019, 2020, 2021). The results from the CS-isomer analysis showed that overall CS-GAG sulfation patterns differed significantly between the two brain regions (*p* < 0.0001) (Figure 6C). The WFA^+^ PNN-rich somatosensory cortex exhibited a 2.4-fold increase in the WFA-recognized non-sulfated CS-O (0S) isomer (*p* < 0.0001) and corresponding 4.2% decrease in CS-A (4S) (*p* < 0.0001), 53.7% decrease in CS-C (6S) (*p* < 0.0001), and 30.2% decrease in CS-D (2S6S) (*p* = 0.0069) isomers compared to the adjacent hippocampus from the same tissue section (Table 3). CS-GAG sulfation patterns between male and female subregions were not significantly different [SCtx (*p* = 0.98), Hip (*p* = 0.09)] (Supplementary Methods). The changes in CS isomer composition identified between these two brain regions also demonstrate hypersulfation of the hippocampal CS-GAGs compared to the somatosensory cortex (*p* < 0.0001), consistent with the significant increase in the average number of sulfates per CS isomer (Figure 6D). These results provide both a correlation between the efficiency of WFA to label PNNs in CS-O (0S)-rich regions of the brain and compelling new evidence supporting the existence of interregional CS-GAG sulfation patterning differences throughout non-diseased brain.

**Table 3:** Interregional changes in CS-GAG sulfation patterns in mouse brain.

	**Cortex % isomer (SE)**	**Hippocampus % isomer (SE)**	**Difference of means**	**95% CI of difference**	***P_***adj***_***
**Male and female (*****n*** **=** **15)**					
CS-O (0S)	16.9 (0.3)	7.1 (0.1)	9.9	9.1 to 10.7	<0.0001
CS-A (4S)	74.9 (0.4)	78.1 (0.2)	−3.2	−4.0 to −2.4	<0.0001
CS-C (6S)	4.9 (0.1)	10.6 (0.1)	−5.7	−6.5 to −4.9	<0.0001
CS-D (2S6S)	2.3 (<0.1)	3.3 (0.1)	−1.0	−1.8 to −0.2	0.0069
CS-E (4S6S)	0.9 (<0.1)	0.9 (<0.1)	<0.1	−0.8 to 0.8	>0.9999
**Male only (*****n*** **=** **8)**					
CS-O (0S)	16.9 (0.4)	7.1 (0.2)	9.8	8.8 to 10.8	<0.0001
CS-A (4S)	74.8 (0.6)	77.8 (0.4)	−3.0	−4.0 to −2.0	<0.0001
CS-C (6S)	5.0 (0.1)	10.8 (0.2)	−5.8	−6.8 to −4.8	<0.0001
CS-D (2S6S)	2.4 (0.1)	3.4 (0.1)	−1.0	−2.0 to <0.1	0.0623
CS-E (4S6S)	0.9 (<0.1)	0.9 (0.1)	<0.1	−1.1 to 1.0	>0.9999
**Female only (*****n*** **=** **7)**					
CS-O (0S)	17.0 (0.5)	7.0 (0.1)	10.0	9.0 to 10.9	<0.0001
CS-A (4S)	75.0 (0.6)	78.5 (0.2)	−3.5	−4.5 to −2.5	<0.0001
CS-C (6S)	4.8 (0.1)	10.3 (0.1)	−5.6	−6.5 to −4.6	<0.0001
CS-D (2S6S)	2.3 (<0.1)	3.3 (0.1)	−1.0	−2.0 to < −0.1	0.0396
CS-E (4S6S)	0.9 (<0.1)	0.9 (<0.01)	0.1	−0.9 to 1.1	>0.9998

*CS, chondroitin sulfate; SE, standard error; CI, confidence interval. Stats: repeated measures using two-way ANOVA with matched CS isomers and matched brain regions, corrected using Šidák's multiple comparisons. % isomer represents the relative abundance of each CS isomer (equal to 100%)*.

## Re-evaluation of PNN “Loss” by WFA Labeling

We now raise the intriguing possibility that the historical use of WFA to label PNNs may have resulted in a significant underestimation of the number and prevalence of these matrices throughout the brain. In support of this theory are data showing (1) WFA lectin preferentially binds to the non-sulfated CS-O (0S) isomer (Figure 4), (2) overexpression of the CS-C (6S) unit reduces both abundance of the CS-O (0S) isomer and WFA labeling of PNNs (Figure 5), and (3) brain regions that exhibit high WFA^+^ PNN labeling also exhibit elevated CS-O (0S) isomer expression (Figure 6). Collectively, these findings now beg the provocative question of whether PNNs are truly “lost” in scenarios of reduced WFA^+^ PNN labeling?

### Reconsideration of PNN “Loss” in AD and Other Neurocognitive Disorders

As aforementioned, loss of WFA^+^ PNNs has been described as a pathological event associated with AD in both humans and mouse models of this disease (Tables 1, 2). While we acknowledge PNN changes may represent an important factor underlying AD pathogenesis, we also now hypothesize that these changes may be mediated through alterations in CS-GAG sulfation patterns (and thereby functioning of the PNNs) instead of overt matrix disassembly. Specifically, we predict that the loss of WFA labeling in postmortem AD brain tissue (Kobayashi et al., 1989; Baig et al., 2005) is primarily driven by reduced recognition of WFA to the hypersulfated CS-GAGs (Logsdon et al., 2021) rather than a reduction in the matrix assembly itself. Since CS-GAG hypersulfation was evident in Braak Stage III/IV control patients prior to the development of AD clinicopathology (Figure 3; see bib37), we hypothesize that a subpopulation of control patients undiagnosed with AD may also exhibit reduced WFA^+^ PNN labeling in association with changes in their underlying CS isomers. Such a possibility provides a reasonable explanation for differences reported in histological PNN changes in human postmortem AD brain tissue between published studies, which we predict may be due to variations in CS-GAG sulfation patterning in both the “non-AD” control population and the extent of which these changes occur in the “AD” population.

The direct influence that CS-GAG sulfation patterning has on histological PNN labeling also provides possible new insights for the interpretation of studies that show the selective accumulation of pTau in non PNN-enmeshed neurons (Figure 2C; see Morawski et al., 2010a); we now ask another provocative question of whether these neurons are truly devoid of PNNs, or are they enmeshed by PNNs of altered protein and glycan composition? If the former, then the current theory that PNNs are “protective” against pTau seeding stands true, but if the latter, then one can then now predict that changes in PNN subpopulations may *enhance* pTau accumulation, potentially providing novel insights into how specific neuronal populations are targeted in the development of AD pathology.

To date, there have been no reports describing changes of brain CS-GAG sulfation patterns in mouse models of AD neuropathology, which we predict will lend valuable information linking CS isomer contribution and accumulation of AD pathology in these models. However, we recently reported that a mouse model of traumatic brain injury and neuroinflammation showed a significant decrease in the CS-O (0S) isomer and increase in the mono-sulfated CS-C (6S) isomer in association with the loss of WFA^+^ PNNs and increased gliosis (Alonge et al., 2021), similar to that observed in human postmortem AD brain tissue (Logsdon et al., 2021). It is possible that neuroinflammation mediates the histochemical “loss” of WFA^+^ PNNs by influencing the PNN CS-GAG composition and reducing the abundance of WFA-recognized CS-O (0S) unit. This possibility aligns with studies that show that microglia inhibition results in increased WFA^+^ PNN histology in normal mice (Liu et al., 2021; Barahona et al., 2022), although whether this effect also associates with changes in the CS-GAG composition remains unknown.

Schizophrenia (SZ) (Mauney et al., 2013; Berretta et al., 2015; Enwright et al., 2016) and bipolar disorder (Alcaide et al., 2019) are two additional neurocognitive diseases associated with changes in PNN matrices. Similar to findings in the AD brain, postmortem brain tissue from SZ patients exhibit marked changes in PNN structures including decreased WFA^+^ PNNs in the amygdala, entorhinal cortex, and pre-frontal cortex (Pantazopoulos et al., 2010; Mauney et al., 2013). As observed in AD, the magnitude of the reduction in PNN glycans appears to be much greater than the associated aggrecan core protein (Pantazopoulos et al., 2015; Enwright et al., 2016), which agrees with recent findings for decreased expression of CS-GAG biosynthesis genes but increased CSPG core protein expression in postmortem SZ human brain tissue (Pantazopoulos et al., 2021). Although the disproportionate loss of CS-GAGs relative to the underlying core protein may contribute to the disease by destabilizing synaptic connectivity, we also cannot dismiss the possibility such observations reflect changes in the underlying PNN species or alterations in the CS-GAG sulfation patterns rather than loss of the matrix itself. We now provide a cautionary note for the interpretation of prior studies that rely on solely on WFA histological labeling to assess PNN abundance without addressing potential changes in the CSPG variant or that of the associated CS-GAG composition.

### Reconsideration of Species-Specific WFA^+^ PNN Labeling

**Figure 7:**
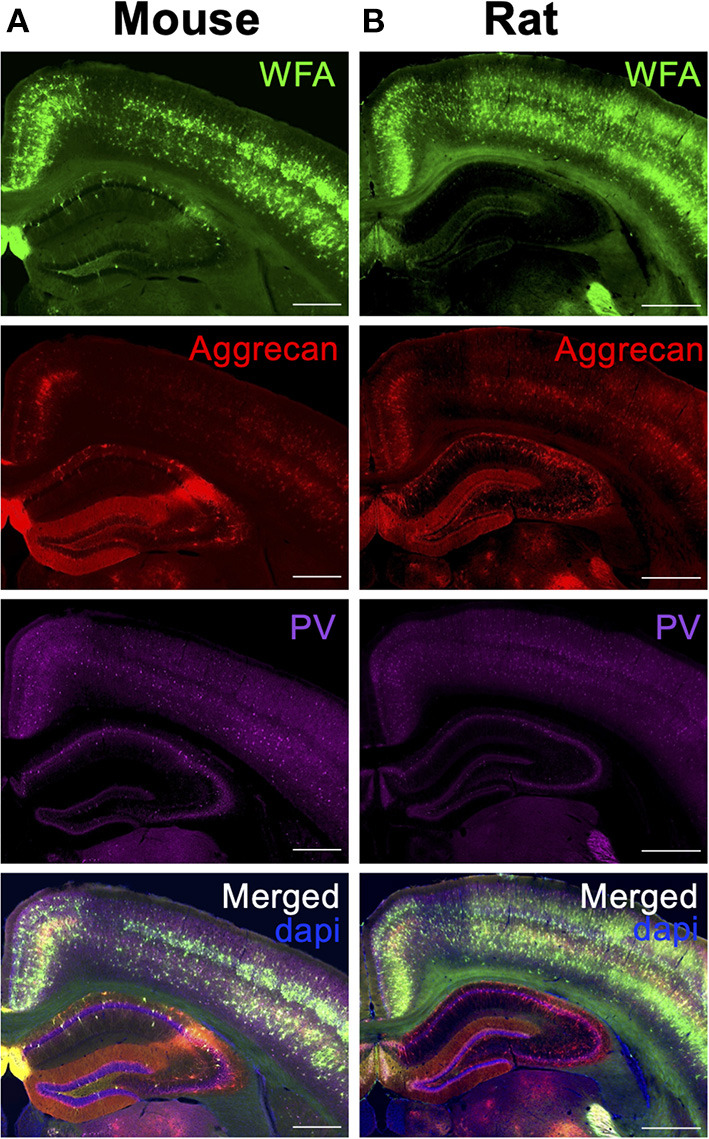
Differences in hippocampal PNN labeling between species. Coronal sections of the dorsal hippocampus for wild type **(A)** mouse and **(B)** rats were labeled for PNN glycans (WFA, *green*) and PNN CSPGs (aggrecan, *red*) and co-localized with GABAergic Parvalbumin (PV, *purple*) neurons. Scale: **(A)** 0.5 mm, **(B)** 1 mm. WFA, *Wisteria floribunda* agglutinin; PV, parvalbumin.

Notable differences between hippocampal PNN subtypes have been reported between mouse and rat hippocampi (Lensjø et al., 2017). In mice, WFA^+^ and aggrecan^+^ PNNs are present throughout all hippocampal subregions (i.e., CA1, CA2, CA3, DG) and in the adjacent cortical regions, with many PNNs exhibiting positive labeling of both targets (Figure 7A; Supplementary Methods). This spatial distribution of PNNs in the mouse brain is in sharp contrast with that observed in the rat brain; here, the rat brain exhibits similar WFA^+^ and aggrecan^+^ PNNs in the cortex but only selectively expresses aggrecan^+^ PNNs in the hippocampus (Figure 7B). We now consider whether reduced labeling of PNNs by WFA specific to the rat hippocampus could either be due to (1) reduced glycosylation and CS-glycan attachment of the aggrecan core protein (Matthews et al., 2002), or (2) changes in the attached PNN CS-GAG composition that favors reduced WFA binding (Nadanaka et al., 2020), the outcome of which greatly influences the functional interpretation of these findings. If the former (reduced glycosylation of CSPGs), then we would predict the rat hippocampus exhibits *decreased* glycan-protein interactions compared to the mouse. However, if the latter (reduced WFA labeling resulting from CS-GAG hypersulfation), then we predict the rat hippocampus would exhibit *increased* glycan-protein interactions. Additional characterization of potential mechanisms underlying these changes, including mass spectrometry of the CS-GAG sulfation patters and labeling using a variety of glycosylated and non-glycosylated aggrecan targeted antibodies (see section PNN Identification Beyond WFA Labeling), are required to resolve this question.

### PNN Identification Beyond WFA Labeling

**Figure 8:**
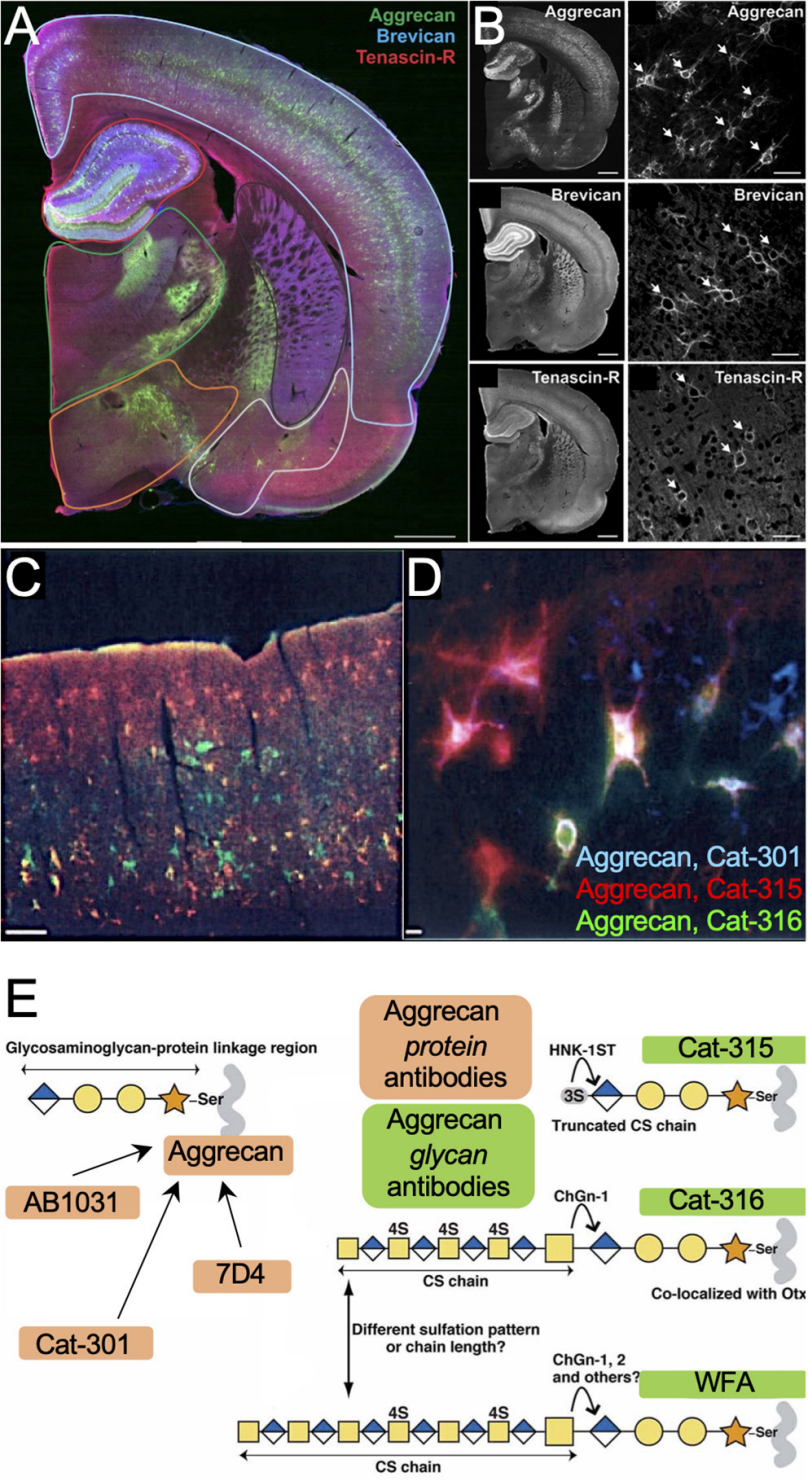
Distribution of PNN subpopulations throughout rat brain. **(A)** Rat brain stained for PNN CSPGs aggrecan (*green*) and brevican (*blue*), and PNN cross-linker Tenascin-R (*red*), shows region-specific differences in **(B)** PNN subpopulations throughout the brain. Scale: 1 mm and 50 μm (See Dauth et al., 2016). **(C,D)** Rat brain labeled for aggrecan^+^ PNNs using antibodies Cat-301 (*blue*), Cat-315 (*red*), and Cat-316 (*green*) recognize different subsets of aggrecan^+^ PNNs in the **(C)** cortex and **(D)** hippocampus. Scale: **(C)** 200 μm, **(D)** 20 μm (see Matthews et al., 2002) Copyright (2002) Society for Neuroscience. **(E)** Variability in aggrecan^+^ PNN labeling may stem from distinctive antibody targets; whereas antibodies AB1031, Cat-301 and 7D4 recognize the aggrecan core protein, antibodies Cat-315, Cat-316 and WFA recognize the attached CS-GAGs and oligosaccharides (see Miyata et al., 2018). WFA, *Wisteria floribunda* agglutinin.

As a first step to concisely address whether PNNs are altered across species, brain regions and in disease states, one should consider labeling for the underlying PNN CSPG core proteins (Figure 1) in addition to WFA lectin labeling of the attached CS-GAGs before determining whether PNNs are “lost” by histochemistry analysis alone. Notably, PNN CSPGs and associated structural glycoproteins exhibit region-specific expression differences throughout the brain. A recent study by Dauth and colleagues showed region-dependent differences in PNN assembly based on the expression of the underlying PNN core proteins (i.e., aggrecan, brevican, TnR) throughout the adult mouse brain (Figure 8A; see Dauth et al., 2016). The positive identification of both brevican^+^ and TnR^+^ PNN matrices in brain regions absent of aggrecan (Figure 8B; see Dauth et al., 2016) provides evidence that brevican^+^ PNN CSPG subpopulations exist independent of aggrecan^+^ PNN CSPG expression. Since the aggrecan core protein also associates with the most CS-GAG attachments (Figure 1), reduction in both aggrecan^+^ and WFA^+^ PNNs is thus insufficient to establish whether all PNNs matrices are “lost” under certain conditions when considering the histological loss aggrecan^+^ and WFA^+^ PNNs may simply result from a compositional shift in the underlying core protein expression. As aforementioned, spatiotemporal shifts in CSPG expression is commonly observed throughout neurodevelopment; whereas mouse brain expression for aggrecan, brevican and versican increase >2-fold between p14 and adulthood, neurocan core protein expression decreases >50% during this time (Carulli et al., 2010). We predict that changes in PNN CSPG core protein expression can occur during disease states independent of normal aging and that this effect would be capable of influencing both aggrecan and WFA histological labeling of PNNs independent to changes in total matrix abundance.

However, we caution that labeling for a single CSPG may yield significantly different results based on the primary antibody chosen for PNN labeling, independent of the core protein expression itself. Aggrecan^+^ PNN staining has been described using multiple different primary antibodies including (but not limited to) AB1031, 7D4, Cat-301, Cat-315, Cat-316, and WFA (Matthews et al., 2002; Virgintino et al., 2009; Miyata et al., 2018; Ueno et al., 2018), which recognize and label distinct subsets of aggrecan^+^ PNNs throughout the brain (Figures 8C,D; see Matthews et al., 2002). Whereas the AB1031, 7D4, and Cat-301 recognize the aggrecan core protein, Cat-315, Cat-316, and WFA recognize CS oligosaccharides on aggrecan (Figure 8E; see Miyata et al., 2018). The glycosylation microheterogeneity of this single CSPG provides fine-tuned temporal spatial regulation, including time, regional, laminar, and cell-type specific expression, all of which are susceptible to changes in normal and neuro-diseased states.

## Limitations, Future Directions, and Conclusions

We previously suggested coupling mass spectrometry analysis of the CS-GAG sulfation patters with histochemical labeling of CSPGs to fully determine whether brain PNN matrix abundance is changing. However, we address a critical limitation for brain CS glycan analysis using mass spectrometry in the absence of buffer fractionation; although mass spectrometry can accurately determine the abundance of each CS isomer from small quantities of tissue, it cannot distinguish between interstitial and PNN sourced glycans. Therefore, a shift in CS-GAG sulfation patterns may in part reflect changes in interstitial matrices, such as that produced by glial scarring, in addition to that of PNNs themselves. Although often seen as independent, we predict that both interstitial and PNN-based CS-GAG matrices are variations of the same pericellular coat capable of enmeshing and interacting with the underlying neuron, and that the glycan-protein interactions observed in one matrix subtype may be applicable to the other. Indeed, not only do PNNs exhibit both diffuse and structured morphologies in the brain (Figure 1), indicating PNN subtypes exist within a “spectrum” of diffuse/interstitial and structured subtypes, but CSPGs primarily found in PNNs (e.g., aggrecan) are also abundant in the interstitial matrix (Deepa et al., 2006). A clear example showcasing this range of interstitial and structured PNN subtypes within the same brain region can be observed in the hippocampus (Figure 7); here, structured-like aggrecan^+^ PNNs are present in both CA1 and CA3 subregions but diffuse/interstitial-like PNNs are dominant in the CA2 and DG. Although these PNN matrices exhibit distinctive region-dependent morphologies, they are all widely considered to be PNNs. The possibility that pericellular coats comprised of both diffuse/interstitial and structured CS-GAG^+^ matrices are capable of exhibiting similar biological functions challenges traditional views of perineuronal nets but allows for a more inclusive outlook on the function of matrices in both health and disease.

**Figure 9:**
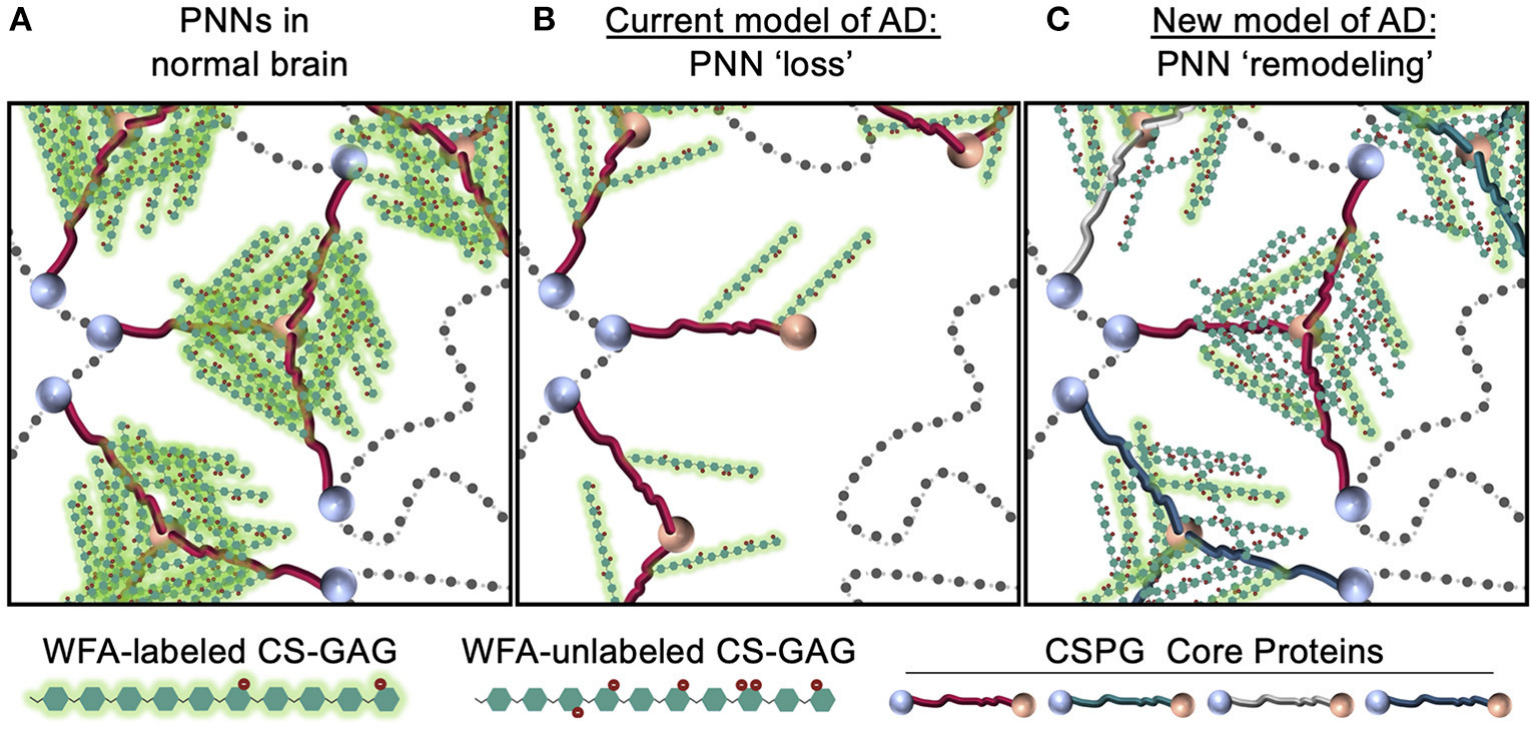
Models depicting PNN “loss” in AD. In contrast to **(A)** normal brain, which contains stable WFA^+^ PNN structures, the **(B)** current model describing PNN “loss” in AD proposes that this neurological disease associates with full degradation of the PNN matrix. In response to current evidence that show postmortem AD brain tissue correlates with increased PNN core protein expression and changes in CS-GAG sulfation patterns, we provide **(C)** an updated model describing PNN “remodeling” as a core feature of the AD brain. This remodeling event associates with a decrease in WFA^+^ PNNs not through matrix degradation but instead through changes in WFA lectin recognition of the hypersulfated diseased CS-GAGs. This new model also considers AD-driven changes in CSPG core protein variants that may support fewer CS-GAG glycan attachments. WFA, *Wisteria floribunda* agglutinin; PNNs, perineuronal nets; CS-GAG, chondroitin sulfate-glycosaminoglycan; CSPG, chondroitin sulfate proteoglycan.

Overall, PNN remodeling may represent an important step in the pathophysiology of AD and other neurocognitive disorders. Changes to PNN structure and function can result from either shifts in core protein variants or sulfation modifications to the attached CS-GAG chains. In this hypothesis and theory article, we have provided ample evidence to support a novel theory that the observed histochemical “loss” of WFA^+^ PNNs in postmortem AD brain tissue and in rodent models of AD neuropathology may be largely driven by WFA's reduced binding to sulfated CS isomers independent of PNN disassociation (Figure 9). Moreover, we propose that the loss of WFA labeling in the absence of core protein changes may be used as an early indicator of PNN CS-GAG sulfation changes, although we caution that additional analytical studies would be required to confirm this possibility. The potential that PNNs are not lost, but instead remodeled, in AD and other neurocognitive disorders would monumentally shift the focus of future studies from PNN degradation and matrix loss to PNN turnover and matrix reassembly (Figure 9). The role for microglia in regulating PNN formation, observed in both normal and diseased brains, suggest that this glia cell type may be involved in maintaining normal diurnal PNN turnover (Pantazopoulos et al., 2020; Barahona et al., 2022) and also may play a role in accelerating the PNN remodeling observed in diseased brains.

## Data Availability Statement

The original contributions presented in the study are included in the article/Supplementary Material, further inquiries can be directed to the corresponding author.

## Ethics Statement

The animal study was reviewed and approved by Institutional Animal Care and Use Committee (IACUC) at the University of Washington (Seattle, Washington).

## Author Contributions

KA, JS, and SH contributed to the manuscript preparation. Tissue immunohistochemistry and analyses were completed by KA and SH. Glycan isolations and mass spectrometry analyses were performed and evaluated by KA. All authors contributed to the article and approved the submitted version.

## Funding

This work was supported by the National Institute of Diabetes and Digestive and Kidney Diseases (NIH-NIDDK) grants DK017047 (KA), DK114474 (JS), and DK128383 (JS). National Institute on Aging (NIH-NIA) grants AG066509 (KA) and AG074152 (KA). Department of Defense grant W81XWH2110635 (JS).

## Conflict of Interest

The authors declare that the research was conducted in the absence of any commercial or financial relationships that could be construed as a potential conflict of interest.

## Publisher's Note

All claims expressed in this article are solely those of the authors and do not necessarily represent those of their affiliated organizations, or those of the publisher, the editors and the reviewers. Any product that may be evaluated in this article, or claim that may be made by its manufacturer, is not guaranteed or endorsed by the publisher.

## References

[B1] Ajmo J. M. Bailey L. A. Howell M. D. Cortez L. K. Pennypacker K. R. Mehta H. N.. (2010). Abnormal post-translational and extracellular processing of brevican in plaque-bearing mice over-expressing APPsw. J. Neurochem. 113, 784–795. 10.1111/j.1471-4159.2010.06647.x 20180882 PMC2855738

[B2] Alcaide J. Guirado R. Crespo C. Blasco-Ibáñez J. M. Varea E. Sanjuan J.. (2019). Alterations of perineuronal nets in the dorsolateral prefrontal cortex of neuropsychiatric patients. Int. J. Bipolar Disord. 7, 24. 10.1186/s40345-019-0161-0 31728775 PMC6856240

[B3] Alonge K. M. Herbert M. J. Yagi M. Cook D. G. Banks W. A. Logsdon A. F. (2021). Changes in brain matrix glycan sulfation associate with reactive gliosis and motor coordination in mice with head trauma. Front. Behav. Neurosci. 15, 745288. 10.3389/fnbeh.2021.745288 34776892 PMC8581466

[B4] Alonge K. M. Logsdon A. F. Murphree T. A. Banks W. A. Keene C. D. Edgar J. S.. (2019). Quantitative analysis of chondroitin sulfate disaccharides from human and rodent fixed brain tissue by electrospray ionization-tandem mass spectrometry. Glycobiology 29, 847–860. 10.1093/glycob/cwz060 31361007 PMC6861844

[B5] Alonge K. M. Mirzadeh Z. Scarlett J. M. Logsdon A. F. Brown J. M. Cabrales E.. (2020). Hypothalamic perineuronal net assembly is required for sustained diabetes remission induced by fibroblast growth factor 1 in rats. Nat. Metab. 2, 1025–1033. 10.1038/s42255-020-00275-6 32895577 PMC7572652

[B6] Ariga T. Miyatake T. Yu R. K. (2010). Role of proteoglycans and glycosaminoglycans in the pathogenesis of Alzheimer's disease and related disorders: amyloidogenesis and therapeutic strategies–a review. J. Neurosci. Res. 88, 2303–2315. 10.1002/jnr.22393 20623617

[B7] Baig S. Wilcock G. K. Love S. (2005). Loss of perineuronal net N-acetylgalactosamine in Alzheimer's disease. Acta Neuropathol. 110, 393–401. 10.1007/s00401-005-1060-2 16133543

[B8] Barahona R. A. Morabito S. Swarup V. Green K. N. (2022). Cortical diurnal rhythms remain intact with microglial depletion. Sci. Rep. 12, 114–114. 10.1038/s41598-021-04079-w 34997092 PMC8742049

[B9] Bartolini B. Thelin M. A. Rauch U. Feinstein R. Oldberg A. Malmstrom A.. (2012). Mouse development is not obviously affected by the absence of dermatan sulfate epimerase 2 in spite of a modified brain dermatan sulfate composition. Glycobiology 22, 1007–1016. 10.1093/glycob/cws065 22496542

[B10] Berretta S. Pantazopoulos H. Markota M. Brown C. Batzianouli E. T. (2015). Losing the sugar coating: potential impact of perineuronal net abnormalities on interneurons in schizophrenia. Schizophr. Res. 167, 18–27. 10.1016/j.schres.2014.12.040 25601362 PMC4504843

[B11] Braak H. Braak E. (1991). Neuropathological stageing of Alzheimer-related changes. Acta Neuropathol. 82, 239–259. 10.1007/BF00308809 1759558

[B12] Brückner G. Hausen D. Härtig W. Drlicek M. Arendt T. Brauer K. (1999). Cortical areas abundant in extracellular matrix chondroitin sulphate proteoglycans are less affected by cytoskeletal changes in Alzheimer's disease. Neuroscience 92, 791–805. 10.1016/S0306-4522(99)00071-8 10426522

[B13] Carulli D. Pizzorusso T. Kwok J. C. Putignano E. Poli A. Forostyak S.. (2010). Animals lacking link protein have attenuated perineuronal nets and persistent plasticity. Brain 133, 2331–2347. 10.1093/brain/awq145 20566484

[B14] Carulli D. Verhaagen J. (2021). An extracellular perspective on CNS maturation: perineuronal nets and the control of plasticity. Int. J. Mol. Sci. 22, 2434. 10.3390/ijms22052434 33670945 PMC7957817

[B15] Cattaud V. Bezzina C. Rey C. C. Lejards C. Dahan L. Verret L. (2018). Early disruption of parvalbumin expression and perineuronal nets in the hippocampus of the Tg2576 mouse model of Alzheimer's disease can be rescued by enriched environment. Neurobiol. Aging 72, 147–158. 10.1016/j.neurobiolaging.2018.08.024 30273829

[B16] Crapser J. D. Spangenberg E. E. Barahona R. A. Arreola M. A. Hohsfield L. A. Green K. N. (2020). Microglia facilitate loss of perineuronal nets in the Alzheimer's disease brain. eBioMedicine 58:102919. 10.1016/j.ebiom.2020.102919 32745992 PMC7399129

[B17] Dauth S. Grevesse T. Pantazopoulos H. Campbell P. H. Maoz B. M. Berretta S.. (2016). Extracellular matrix protein expression is brain region dependent. J. Comp. Neurol. 524, 1309–1336. 10.1002/cne.23965 26780384 PMC7714387

[B18] Deepa S. S. Carulli D. Galtrey C. Rhodes K. Fukuda J. Mikami T.. (2006). Composition of perineuronal net extracellular matrix in rat brain: a different disaccharide composition for the net-associated proteoglycans. J. Biol. Chem. 281, 17789–17800. 10.1074/jbc.M600544200 16644727

[B19] Dewitt D. A. Silver J. Canning D. R. Perry G. (1993). Chondroitin sulfate proteoglycans are associated with the lesions of Alzheimer's disease. Exp. Neurol. 121, 149–152. 10.1006/exnr.1993.1081 8339766

[B20] Djerbal L. Lortat-Jacob H. Kwok J. (2017). Chondroitin sulfates and their binding molecules in the central nervous system. Glycoconj. J. 34, 363–376. 10.1007/s10719-017-9761-z 28101734 PMC5487772

[B21] Enwright J. F. Sanapala S. Foglio A. Berry R. Fish K. N. Lewis D. A. (2016). Reduced labeling of parvalbumin neurons and perineuronal nets in the dorsolateral prefrontal cortex of subjects with schizophrenia. Neuropsychopharmacology 41, 2206–2214. 10.1038/npp.2016.24 26868058 PMC4946056

[B22] Foscarin S. Raha-Chowdhury R. Fawcett J. W. Kwok J. C. F. (2017). Brain ageing changes proteoglycan sulfation, rendering perineuronal nets more inhibitory. Aging 9, 1607–1622. 10.18632/aging.101256 28657900 PMC5509459

[B23] Gilbert R. J. Mckeon R. J. Darr A. Calabro A. Hascall V. C. Bellamkonda R. V. (2005). CS-4,6 is differentially upregulated in glial scar and is a potent inhibitor of neurite extension. Mol. Cell. Neurosci. 29, 545–558. 10.1016/j.mcn.2005.04.006 15936953

[B24] Golgi C. (1898). Intomo alla struttura delle cellule nervose. Boll. Soc. Med. Chir. Pavia 1, 1–14.

[B25] Grothe M. J. Sepulcre J. Gonzalez-Escamilla G. Jelistratova I. Schöll M. Hansson O.. (2018). Molecular properties underlying regional vulnerability to Alzheimer's disease pathology. Brain 141, 2755–2771. 10.1093/brain/awy189 30016411 PMC6113636

[B26] Härtig W. Derouiche A. Welt K. Brauer K. Grosche J. Mäder M.. (1999). Cortical neurons immunoreactive for the potassium channel Kv3.1b subunit are predominantly surrounded by perineuronal nets presumed as a buffering system for cations. Brain Res. 842, 15–29. 10.1016/S0006-8993(99)01784-9 10526091

[B27] Hilbig H. Bidmon H. J. Blohm U. Zilles K. (2001). Wisteria floribunda agglutinin labeling patterns in the human cortex: a tool for revealing areal borders and subdivisions in parallel with immunocytochemistry. Anat. Embryol. 203, 45–52. 10.1007/s004290000135 11195088

[B28] Howell M. D. Bailey L. A. Cozart M. A. Gannon B. M. Gottschall P. E. (2015). Hippocampal administration of chondroitinase ABC increases plaque-adjacent synaptic marker and diminishes amyloid burden in aged APPswe/PS1dE9 mice. Acta Neuropathol. Commun. 3, 54–54. 10.1186/s40478-015-0233-z 26337292 PMC4559967

[B29] Hsiao K. Chapman P. Nilsen S. Eckman C. Harigaya Y. Younkin S.. (1996). Correlative memory deficits, Abeta elevation, and amyloid plaques in transgenic mice. Science 274, 99–102. 10.1126/science.274.5284.99 8810256

[B30] Huynh M. B. Ouidja M. O. Chantepie S. Carpentier G. Maïza A. Zhang G.. (2019). Glycosaminoglycans from Alzheimer's disease hippocampus have altered capacities to bind and regulate growth factors activities and to bind tau. PLoS ONE 14, e0209573. 10.1371/journal.pone.0209573 30608949 PMC6319808

[B31] Javonillo D. I. Tran K. M. Phan J. Hingco E. Kramár E. A. Da Cunha C.. (2021). Systematic phenotyping and characterization of the 3xTg-AD mouse model of Alzheimer's disease. Front. Neurosci. 15, 785276. 10.1101/2021.10.01.462640 35140584 PMC8818877

[B32] Kobayashi K. Emson P. C. Mountjoy C. Q. (1989). Vicia villosa lectin-positive neurones in human cerebral cortex. Loss in Alzheimer-type dementia. Brain Res. 498, 170–174. 10.1016/0006-8993(89)90416-2 2790470

[B33] Lendvai D. Morawski M. Négyessy L. Gáti G. Jäger C. Baksa G.. (2013). Neurochemical mapping of the human hippocampus reveals perisynaptic matrix around functional synapses in Alzheimer's disease. Acta Neuropathol. 125, 215–229. 10.1007/s00401-012-1042-0 22961619 PMC6485544

[B34] Lensjø K. K. Christensen A. C. Tennøe S. Fyhn M. Hafting T. (2017). Differential expression and cell-type specificity of perineuronal nets in hippocampus, medial entorhinal cortex, and visual cortex examined in the rat and mouse. eNeuro 4:ENEURO.0379-16. 10.1523/ENEURO.0379-16.2017 28593193 PMC5461557

[B35] Liu Y.-J. Spangenberg E. E. Tang B. Holmes T. C. Green K. N. Xu X. (2021). Microglia elimination increases neural circuit connectivity and activity in adult mouse cortex. J. Neurosci. 41, 1274–1287. 10.1523/JNEUROSCI.2140-20.2020 33380470 PMC7888230

[B36] Logsdon A. F. Francis K. L. Richardson N. E. Hu S. J. Faber C. L. Phan B. A.. (2021). Decoding perineuronal net glycan sulfation patterns in the Alzheimer's disease brain. Alzheimers Dement. 18, 942–954. 10.1002/alz.12451 34482642 PMC8897514

[B37] Matthews R. T. Kelly G. M. Zerillo C. A. Gray G. Tiemeyer M. Hockfield S. (2002). Aggrecan glycoforms contribute to the molecular heterogeneity of perineuronal nets. J. Neurosci. 22, 7536–7547. 10.1523/JNEUROSCI.22-17-07536.2002 12196577 PMC6757962

[B38] Mauney S. A. Athanas K. M. Pantazopoulos H. Shaskan N. Passeri E. Berretta S.. (2013). Developmental pattern of perineuronal nets in the human prefrontal cortex and their deficit in schizophrenia. Biol. Psychiatry 74, 427–435. 10.1016/j.biopsych.2013.05.007 23790226 PMC3752333

[B39] Mckeon R. J. Jurynec M. J. Buck C. R. (1999). The chondroitin sulfate proteoglycans neurocan and phosphacan are expressed by reactive astrocytes in the chronic CNS glial scar. J. Neurosci. 19, 10778–10788. 10.1523/JNEUROSCI.19-24-10778.1999 10594061 PMC6784959

[B40] Mehta D. Jackson R. Paul G. Shi J. Sabbagh M. (2017). Why do trials for Alzheimer's disease drugs keep failing? A discontinued drug perspective for 2010-2015. Expert Opin. Investig. Drugs 26, 735–739. 10.1080/13543784.2017.1323868 28460541 PMC5576861

[B41] Miyata S. Kitagawa H. (2016). Chondroitin 6-sulfation regulates perineuronal net formation by controlling the stability of aggrecan. Neural Plast. 2016, 1305801. 10.1155/2016/1305801 27057358 PMC4738747

[B42] Miyata S. Komatsu Y. Yoshimura Y. Taya C. Kitagawa H. (2012). Persistent cortical plasticity by upregulation of chondroitin 6-sulfation. Nat. Neurosci. 15, 414–422. 10.1038/nn.3023 22246436

[B43] Miyata S. Nadanaka S. Igarashi M. Kitagawa H. (2018). Structural variation of chondroitin sulfate chains contributes to the molecular heterogeneity of perineuronal nets. Front. Integr. Neurosci. 12:3. 10.3389/fnint.2018.00003 29456495 PMC5801575

[B44] Morawski M. Brückner G. Jäger C. Seeger G. Arendt T. (2010a). Neurons associated with aggrecan-based perineuronal nets are protected against tau pathology in subcortical regions in Alzheimer's disease. Neuroscience 169, 1347–1363. 10.1016/j.neuroscience.2010.05.022 20497908

[B45] Morawski M. Bruckner G. Jager C. Seeger G. Matthews R. T. Arendt T. (2012). Involvement of perineuronal and perisynaptic extracellular matrix in Alzheimer's disease neuropathology. Brain Pathol. 22, 547–561. 10.1111/j.1750-3639.2011.00557.x 22126211 PMC3639011

[B46] Morawski M. Pavlica S. Seeger G. Grosche J. Kouznetsova E. Schliebs R.. (2010b). Perineuronal nets are largely unaffected in Alzheimer model Tg2576 mice. Neurobiol. Aging 31, 1254–1256. 10.1016/j.neurobiolaging.2008.07.023 18829133

[B47] Nadanaka S. Miyata S. Yaqiang B. Tamura J.-I. Habuchi O. Kitagawa H. (2020). Reconsideration of the semaphorin-3A binding motif found in chondroitin sulfate using galnac4s-6st-knockout mice. Biomolecules 10, 1499. 10.3390/biom10111499 33143303 PMC7694144

[B48] Nicholson C. Syková E. (1998). Extracellular space structure revealed by diffusion analysis. Trends Neurosci. 21, 207–215. 10.1016/S0166-2236(98)01261-2 9610885

[B49] Pantazopoulos H. Gisabella B. Rexrode L. Benefield D. Yildiz E. Seltzer P.. (2020). Circadian rhythms of perineuronal net composition. eneuro 7, ENEURO.0034-0019.2020. 10.1523/ENEURO.0034-19.2020 32719104 PMC7405073

[B50] Pantazopoulos H. Katsel P. Haroutunian V. Chelini G. Klengel T. Berretta S. (2021). Molecular signature of extracellular matrix pathology in schizophrenia. Eur. J. Neurosci. 53, 3960–3987. 10.1111/ejn.15009 33070392 PMC8359380

[B51] Pantazopoulos H. Markota M. Jaquet F. Ghosh D. Wallin A. Santos A.. (2015). Aggrecan and chondroitin-6-sulfate abnormalities in schizophrenia and bipolar disorder: a postmortem study on the amygdala. Transl. Psychiatry 5,–e496. 10.1038/tp.2014.128 25603412 PMC4312825

[B52] Pantazopoulos H. Woo T. U. Lim M. P. Lange N. Berretta S. (2010). Extracellular matrix-glial abnormalities in the amygdala and entorhinal cortex of subjects diagnosed with schizophrenia. Arch. Gen. Psychiatry 67, 155–166. 10.1001/archgenpsychiatry.2009.196 20124115 PMC4208310

[B53] Reichelt A. C. (2020). Is loss of perineuronal nets a critical pathological event in Alzheimer's disease? eBioMedicine 59:102946. 10.1016/j.ebiom.2020.102946 32810826 PMC7452426

[B54] Rey C. C. Robert V. Bouisset G. Loisy M. Lopez S. Cattaud V.. (2022). Altered inhibitory function in hippocampal CA2 contributes in social memory deficits in Alzheimer's mouse model. iScience 25, 103895–103895. 10.1016/j.isci.2022.103895 35243253 PMC8873612

[B55] Seeger G. Lüth H. J. Winkelmann E. Brauer K. (1996). Distribution patterns of Wisteria floribunda agglutinin binding sites and parvalbumin-immunoreactive neurons in the human visual cortex: a double-labelling study. J. Hirnforsch. 37, 351–366. 8872558

[B56] Shida M. Mikami T. Tamura J. I. Kitagawa H. (2019). Chondroitin sulfate-D promotes neurite outgrowth by acting as an extracellular ligand for neuronal integrin αVβ3. Biochim. Biophys. Acta Gen. Subj. 1863, 1319–1331. 10.1016/j.bbagen.2019.06.004 31181256

[B57] Shimizu H. Ghazizadeh M. Sato S. Oguro T. Kawanami O. (2009). Interaction between β-amyloid protein and heparan sulfate proteoglycans from the cerebral capillary basement membrane in Alzheimer's disease. J. Clin. Neurosci. 16, 277–282. 10.1016/j.jocn.2008.04.009 19091577

[B58] Smith P. D. Coulson-Thomas V. J. Foscarin S. Kwok J. C. F. Fawcett J. W. (2015). “GAG-ing with the neuron”: the role of glycosaminoglycan patterning in the central nervous system. Exp. Neurol. 274, 100–114. 10.1016/j.expneurol.2015.08.004 26277685

[B59] Sorg B. A. Berretta S. Blacktop J. M. Fawcett J. W. Kitagawa H. Kwok J. C. F.. (2016). Casting a wide net: role of perineuronal nets in neural plasticity. J. Neurosci. 36, 11459. 10.1523/JNEUROSCI.2351-16.2016 27911749 PMC5125213

[B60] Sos K. E. Mayer M. I. Takács V. T. Major A. Bardóczi Z. Beres B. M.. (2020). Amyloid β induces interneuron-specific changes in the hippocampus of APPNL-F mice. PLoS ONE 15, e0233700. 10.1371/journal.pone.0233700 32469963 PMC7259556

[B61] Syková E. Nicholson C. (2008). Diffusion in brain extracellular space. Physiol. Rev. 88, 1277–1340. 10.1152/physrev.00027.2007 18923183 PMC2785730

[B62] Testa D. Prochiantz A. Di Nardo A. A. (2019). Perineuronal nets in brain physiology and disease. Semin. Cell Dev. Biol. 89, 125–135. 10.1016/j.semcdb.2018.09.011 30273653

[B63] Ueno H. Fujii K. Suemitsu S. Murakami S. Kitamura N. Wani K.. (2018). Expression of aggrecan components in perineuronal nets in the mouse cerebral cortex. IBRO Rep. 4, 22–37. 10.1016/j.ibror.2018.01.002 30135949 PMC6084874

[B64] Vallet S. D. Clerc O. Ricard-Blum S. (2021). Glycosaminoglycan-protein interactions: the first draft of the glycosaminoglycan interactome. J. Histochem. Cytochem. 69, 93–104. 10.1369/0022155420946403 32757871 PMC7841700

[B65] Végh M. J. Heldring C. M. Kamphuis W. Hijazi S. Timmerman A. J. Li K. W.. (2014). Reducing hippocampal extracellular matrix reverses early memory deficits in a mouse model of Alzheimer's disease. Acta Neuropathol. Commun. 2, 76. 10.1186/s40478-014-0076-z 24974208 PMC4149201

[B66] Virgintino D. Perissinotto D. Girolamo F. Mucignat M. T. Montanini L. Errede M.. (2009). Differential distribution of aggrecan isoforms in perineuronal nets of the human cerebral cortex. J. Cell. Mol. Med. 13, 3151–3173. 10.1111/j.1582-4934.2009.00694.x 19220578 PMC4516474

[B67] Vitellaro-Zuccarello L. De Biasi S. Spreafico R. (1998). One hundred years of Golgi's “perineuronal net”: history of a denied structure. Ital. J. Neurol. Sci. 19, 249–253. 10.1007/BF02427613 10933466

[B68] Yamada J. Jinno S. (2017). Molecular heterogeneity of aggrecan-based perineuronal nets around five subclasses of parvalbumin-expressing neurons in the mouse hippocampus. J. Compar. Neurol. 525, 1234–1249. 10.1002/cne.24132 27718219

[B69] Yang S. Cacquevel M. Saksida L. M. Bussey T. J. Schneider B. L. Aebischer P.. (2015). Perineuronal net digestion with chondroitinase restores memory in mice with tau pathology. Exp. Neurol. 265, 48–58. 10.1016/j.expneurol.2014.11.013 25483398 PMC4353684

[B70] Yang S. Gigout S. Molinaro A. Naito-Matsui Y. Hilton S. Foscarin S.. (2021). Chondroitin 6-sulphate is required for neuroplasticity and memory in ageing. Mol. Psychiatry 26, 5658–5668. 10.1038/s41380-021-01208-9 34272488 PMC8758471

