# Supplementary material for: The “Loss” of Perineuronal Nets in Alzheimer's Disease: Missing or Hiding in Plain Sight?
Source: Front Integr Neurosci. 2022 May 25;16:896400. doi: 10.3389/fnint.2022.896400 (PMC9174696; doi:10.3389/fnint.2022.896400)
Supplement: Supplementary file 1 [file Data_Sheet_1.PDF]

frontiers

Supplementary Material

The 'Loss' of Perineuronal Nets in Alzheimer's Disease: Missing or Hiding in Plain Sight?

Jarrad M. Scarlett, Shannon J. Hu, Kimberly M. Alonge

1 Supplementary Methods

1.1. Animals

For experiments investigating interregional changes in CS-GAG sulfation patterns in mice, male (n=8) and female (n=7) mice bred in our colony on the C57Bl/6J background were sacrificed at 42 d of age. For experiments investigating differences in hippocampal PNN labeling between species, 3 month-old C57Bl/6J mice were purchased from Jackson Laboratories and compared to 3 month-old Sprague Dawley rats purchased from Charles River. Mice and rats had *ad libitum* access to chow and water and were group housed based on sex up to n=5 mice/cage or n=2 rats/cage in temperature-controlled rooms under 12h-dark: 12h-light cycles. All procedures were performed in accordance with NIH guidelines for the care and use of laboratory animals and were approved by the Institutional Animal Care and Use Committee (IACUC) at the University of Washington (Seattle, Washington), and all experiments were conducted in accordance with the National Institutes of Health Guide for the Care and Use of Laboratory Animals. All experiments reported are in compliance with ARRIVE guidelines.

1.2. Brain processing

Mice and rats were anesthetized with ketamine and xylazine and cardiac perfused with 0.1 M phosphate buffered saline (PBS) followed by 4% paraformaldehyde (PFA) in 0.1 M PBS. Brains were extracted, post fixed for 48 h in 4% PFA at 4°C, cryopreserved in 30% sucrose in 0.1 M PBS solution, and frozen in optimal cutting temperature (OCT) compound on dry ice. Brains were stored at −80°C degrees until they were ready for cryosectioning, when they were then moved and equilibrated at -20°C 24 hours prior to sectioning. Brains were cut with a Leica CM1950 cryostat at 30 µm-thick serial sections and stored in 0.1 M PBS + 0.02% sodium azide at 4°C as free-floating sections before processing for immunofluorescence and CS-GAG extraction. For the interregional mouse study, brain tissues were collected, processed, and imaged in tandem using the same imaging and quantifications settings for histochemical analysis. For the mouse and rat interspecies study, brains were collected separately, processed together, but imaged separately to produce the representative images.

1.3. Immunofluorescent labeling and wide-field fluorescence microscopy

30 µm-thick coronal sections for mouse and rat underwent antigen retrieval in 10 mM trisodium citrate (pH 8.5) by heating at 90°C for 20 min. Immunostaining of free-floating tissue were performed by permeabilizing the tissue for 30 m at room temperature (RT) in 0.1 M PBS + 0.2% Triton X-100 followed by tissue blocking for 2 h at 37°C in 0.1 M PBS + 0.05% Triton X-100

(PBST) + 10% normal donkey serum (Jackson ImmunoResearch). Sections were then incubated overnight at 4°C using 1:1,000 dilution of biotin labeled *Wisteria floribunda* agglutinin (WFA) (Sigma: L1516) in PBST + 1% donkey serum (for interregional PNN comparisons in mouse tissue) or a mix of 1:1,000 WFA, 1:1,000 aggrecan (Millipore; AB1031), and 1:1,000 parvalbumin (R&D; AF5058) in PBST + 1% donkey serum (for species comparison). The next morning, sections were washed and incubated for 2 h at RT in a 1:1,000 dilution of Alexa-fluor-conjugated secondary antibodies in PBST + 1% donkey serum. Sections were then counterstained for DAPI, mounted, and cover slipped using Fluoromount-G (ThermoFisher, 4958-02). Tile imaging was conducted with a Keyence BZ-800 inverted microscope using dapi, FITC, Cy3, and Cy5 channels at a 10X objective. Images were processed with Fiji open-source imaging software. To quantify the mean fluorescent intensity (MFI) for WFA, we defined a set region of interest (somatosensory cortex and hippocampus; refer to outlines in Figure 6), converted the images to 8-bit, subtracted a constant background, and quantified the MFI normalized to the outlined area (per mm^2).

1.4. Brain CS-GAG digestion and CS disaccharide isolation

10 sections of PFA-fixed coronal brain sections (30 μm) containing both the somatosensory cortex and dorsal hippocampus from each mouse were chosen for CS disaccharide extraction and quantification. Somatosensory cortical and hippocampal regions were isolated using a #10 scalpel and were washed 3x in Optima LC/MS-grade water and 1x in 50 mM ammonium bicarbonate (pH 7.6) at RT. Chondroitinase ABC enzyme (ChABC, Sigma, C3667) was reconstituted (500 mU/mL) in 50 mM ammonium bicarbonate (pH 7.6) to digest CS-GAGs from the fixed brain regions at 37°C in a Thermo Scientific MaxQ4000 orbital shaker for 24 h. Supernatants were collected in sterile 1.7 mL microcentrifuge tubes and spun for 10 m at 14k x g to pellet any debris. The supernatant was then dehydrated using a SpeedVac Concentrator, and the lyophilized product was reconstituted in 30 μL of LC/MS-grade water.

1.5. Mass spectrometry quantification of the relative abundance of CS isomers

Isolated CS samples were analyzed using a triple quadrupole mass spectrometer equipped with an electrospray ion source (Waters Xevo TQ-S) operated in negative mode ionization. Liquid chromatography-tandem mass spectrometry (LC-MS/MS) was performed using a Waters Acquity I-class ultra-performance liquid chromatographic system (UPLC) coupled to the same Waters Xevo TQ-S system. Disaccharides were resolved by porous graphitic chromatography (Hypercarb column; 2.1 x 50 mm, 3 μm; ThermoFisher) and assigned multiple reaction monitoring (MRM) channels: CS-A (4S), *m/z* 458 > 300; CS-C (6S), *m/z* 458 > 282; CS-D (2S6S) and CS-E (4S6S), *m/z* 268 > 282; CS-O (0S), *m/z* 378 > 175. MassLynx software version 4.1 (Waters) was used to acquire and quantify all data. Under the conditions described above, the ratios between peak areas produced from equimolar CS standard runs were normalized to the highest peak intensity and relative quantification of each CS isomer within a sample was achieved using a modified peak area normalization function. Each CS isomer was expressed as a relative abundance percentage of the total CS isomer composition within each sample. Industry-grade CS disaccharide standards CS-A (4S) (CD002), CS-C (6S) (CD003), CS-D (2S6S) (CD006), CS-E (4S6S) (CD004) and CS-O (0S) (CD001) were purchased from Galen Laboratory Supplies (North Haven, CT). Acetonitrile (optima LC/MS-grade) and all other reagents were obtained from Fisher Scientific.

1.6. Statistics

A two-way anova with matched isomers and paired brain regions (for interregional comparisons) and a two-way anova with matched isomers only (for sex differences) fit full interaction term models with multiple comparisons corrected using Šidák test in GraphPad Prism® 8.0 (Graph Pad Software, Inc., La Jolla, CA). Error bars represent the standard error (SE) of the mean.
